# Genome‐wide association study identified novel candidate loci affecting wood formation in Norway spruce

**DOI:** 10.1111/tpj.14429

**Published:** 2019-07-28

**Authors:** John Baison, Amaryllis Vidalis, Linghua Zhou, Zhi‐Qiang Chen, Zitong Li, Mikko J. Sillanpää, Carolina Bernhardsson, Douglas Scofield, Nils Forsberg, Thomas Grahn, Lars Olsson, Bo Karlsson, Harry Wu, Pär K. Ingvarsson, Sven‐Olof Lundqvist, Totte Niittylä, M Rosario García‐Gil

**Affiliations:** ^1^ Department of Forest Genetics and Plant Physiology Umeå Plant Science Centre Swedish University of Agricultural Science Parallellvägen 21 Umeå 907 36 Sweden; ^2^ Section of Population Epigenetics and Epigenomics Centre of Life and Food Sciences Weihenstephan Technische Universität München Lichtenbergstr. 2a München 85748 Germany; ^3^ Ecological Genetics Research Unit Department of Biosciences University of Helsinki P.O. Box 65 FI‐00014 Helsinki Finland; ^4^ Department of Mathematical Sciences Biocenter Oulu University of Oulu Pentti Kaiteran katu 1 Oulu Finland; ^5^ Department of Ecology and Environmental Science Umeå University Linnaeus väg 4-6 Umeå 907 36 Sweden; ^6^ Uppsala Multidisciplinary Centre for Advanced Computational Science Uppsala University Lägerhyddsvägen 2 Uppsala 752 37 Sweden; ^7^ RISE Bioeconomy Drottning Kristinas väg 61 SE‐114 86 Stockholm Sweden; ^8^ Skogforsk Ekebo 2250 SE‐268 90 Svalöv Sweden; ^9^ Department of Ecology and Genetics: Evolutionary Biology Uppsala University Kåbovägen 4 Uppsala 752 36 Sweden; ^10^ IIC Rosenlundsgatan 48B SE‐118 63 Stockholm Sweden

**Keywords:** candidate genes, functional trait mapping, genome‐wide association mapping, Norway spruce, sequence capture, single nucleotide polymorphisms

## Abstract

Norway spruce is a boreal forest tree species of significant ecological and economic importance. Hence there is a strong imperative to dissect the genetics underlying important wood quality traits in the species. We performed a functional genome‐wide association study (GWAS) of 17 wood traits in Norway spruce using 178 101 single nucleotide polymorphisms (SNPs) generated from exome genotyping of 517 mother trees. The wood traits were defined using functional modelling of wood properties across annual growth rings. We applied a  Least Absolute Shrinkage and Selection Operator (LASSO‐based) association mapping method using a functional multilocus mapping approach that utilizes latent traits, with a stability selection probability method as the hypothesis testing approach to determine a significant quantitative trait locus. The analysis provided 52 significant SNPs from 39 candidate genes, including genes previously implicated in wood formation and tree growth in spruce and other species. Our study represents a multilocus GWAS for complex wood traits in Norway spruce. The results advance our understanding of the genetics influencing wood traits and identifies candidate genes for future functional studies.

## Significance Statement

Wood provides both structural support and a transport route for water and solutes in trees. Our work provides a framework to dissect the genetic nature of wood formation and adds to our understanding of tree growth and development. With the current research focus on wood cell wall biosynthesis in general, and lignocellulose feedstock for biorefineries, we believe that this contribution will be of wide interest for the plant science community.

## Introduction

Norway spruce (*Picea abies* (L.) Karst.) is a dominant boreal species of significant economic and ecological importance (Hannrup *et al*., 2004). Long‐term Norway spruce breeding programmes for improvement of growth and survival were initiated in the 1940s and recently, wood quality has become one of the priority traits (Bertaud and Holmbom, 2004; Hannrup *et al*., 2004). Norway spruce breeding in Sweden completes one cycle in about 20 years and such long generation time makes improvements in growth and wood quality very slow. Among wood quality traits, wood density is considered a key indicator of stability, strength and stiffness of sawn timber (Hauksson *et al*., 2001). Several studies of wood quality observed that fast growth conflicts with high quality wood, as shown by the negative genetic correlation between wood volume growth and density in Norway spruce (Olesen, 1977; Dutilleul *et al*., 1998; Chen *et al*., 2014). However in several conifers such as Scots pine (*P. sylvestris* L.) and red pine the relationship has been inconsistent (Larocque and Marshall, 1995; Peltola *et al*., 2009). To combine fast growth and desirable wood properties through breeding, and to shorten the breeding cycle, it is therefore imperative to design effective early selection methods and breeding strategies. In an effort to design optimal breeding and selection strategies it is essential to identify alleles that are responsible for generating favourable or unfavourable genetic correlations (Hallingbäck *et al*., 2014).

One of the early studies in conifers identified quantitative trait locus (QTLs) for wood density variation in loblolly pine using linkage analyses based on segregating family pedigrees (Groover *et al*., 1994). However, marker‐aided selection (MAS) based on results from QTL linkage analyses were never implemented in practical tree breeding due to the so‐called Beavis effect (e.g. inflated estimates of allelic effects and underestimation of QTL number for economically important traits) (Beavis, 1998), inconsistent associations among different families and the low transferability of markers (Strauss *et al*., 1992). Association mapping (AM), also called linkage disequilibrium (LD) mapping, is a powerful alternative QTL detection method that was introduced to tree genetics using a candidate gene approach (Thumma *et al*., 2010). AM overcomes the limited resolution of family‐based QTL linkage mapping by relying on historical recombination in the mapping population (Neale and Savolainen, 2004; Thavamanikumar *et al*., 2013; Huang and Han, 2014). However, the genome‐wide levels of LD in Norway spruce has been revealed to be complex and highly heterogenous (Larsson *et al*., 2013). Therefore, AM is also vulnerable to some confounding historical factors such as population admixture, selection pressures which include possible genetic drift. Therefore, population genetic structure, kinship and LD within the study population need to be carefully accounted for in the analysis to minimize false positives (Khan and Korban, 2012).

The availability of a draft genome sequence for Norway spruce (Nystedt *et al*., 2013) has provided numerous possibilities for the development of genetic markers to conduct both AM at the genome‐wide level (genome‐wide association, GWAS) and genomic selection (GS). Several reduced representation‐based approaches such as sequence capture and transcriptome sequencing (Hirsch *et al*., 2014) have been developed for studying large genomes, such as the 20 Gb Norway spruce genome. These approaches reduce the sequence space by decreasing the repetitive sequence content of the genome.

Several AM studies have been performed on traits in different tree species and have identified genetic loci linked to, for instance, wood properties in *Populus trichocarpa* Torr. & A. Gray ex. Hook (Porth *et al*., 2013) and *Eucalyptus* (Resende *et al*., 2017b), and adaptive traits in *Pinus contorta* Douglas (Parchman *et al*., 2012). Some genes may impact the trait development at a particular developmental stage, whereas others may alter, or control, rates of change and transitions between consecutive stages (Xing *et al*., 2012; Anderegg, 2015). Studies aimed at dissecting the genetic basis of such dynamics in wood properties can benefit from the application of mathematical functions that account for year‐to‐year variation across annual growth rings, cambial age and distance from pith (Li *et al*., 2014). The development of mathematical methods for the analysis of these longitudinal traits has made it possible to map QTLs underlying the dynamics of developmental traits (Yang *et al*., 2006; Li and Sillanpää, 2013; Camargo *et al*., 2018), and to enhance our understanding of the genetic architecture of the growth trajectories of such dynamic traits (Ma *et al*., 2002; Xing *et al*., 2012). Such functional mapping analysis can be conducted using a multistage approach (Heuven and Janss, 2010). First, the phenotype trends of each individual are modelled using curve‐fitting methods and the parameters describing the curve are then considered as latent traits. The latent traits are then used in an independent association analyses to search for genomic regions affecting the trait and to estimate genetic marker effects (Li and Sillanpää, 2013; Li *et al*., 2014).

In this study, we applied a functional AM approach to identify genomic regions contributing to wood quality traits in Norway spruce. We applied spline models since traditional analyses that utilise a single point data across annual growth rings may confound the analyses by averaging across a full sample. Such averaging may obscure mechanisms acting at specific time points during wood formation and will make identification of underlying genes more difficult. This study has performed the analysis of number of cells per ring calculated from SilviScan data. Penalized LASSO regression (Tibshirani, 1996) and the stability selection probability method (Meinshausen and Bühlmann, 2010) were then used, to detect significant associations between latent traits derived from estimated breeding values (EBVs) and 178101 SNP markers covering the Norway spruce genome.

## Results and Discussion

All 517 Norway spruce maternal trees in the study were considered for variant detection and an average of 1.5 million paired‐end reads were sequenced per individual resulting in 178 101 SNPs. Most SNPs were missense (61%). Applying the probability of stability selection (SSP) to the intercept, slope and two nodes (β_2_ and β_3_) we detected 52 significant QTLs in 17 individual traits whose phenotypic variance explained (PVE) ranged from 0.01 to 4.93% (Table 1). 14 of the significant markers were consistent with overdominance (|*d/a*| > 1.25), with the remaining being codominant (27) (|*d/a*| < 0.50) and 10 exhibiting partial to full dominance (0.50 < |*d/a*| < 1.25) (Table 2, Figure 3).

**Table 1:** Phenotypes, latent traits, SNP, SNP feature, frequency and PVE

Phenotype	Latent Trait	QTL	SNP^a^	Allele	SNP Feature	Frequency	PVE (%)
WD	Intercept	167610	MA_10435406_13733	A/G	Downstream variant	0.71	4.64
	Slope	30469	MA_33109_11804	A/G	Upstream variant	0.72	4.50
	β_2_	30469	MA_33109_11804	A/G	Upstream variant	0.551	4.15
	β_3_	157442	MA_10432646_63090	G/A	Upstream variant	0.567	2.43
EWD	Intercept	167610	MA_10435406_13733	A/G	Downstream variant	0.545	3.38
	Slope	23798	MA_20321_44812	C/T	Upstream variant	0.53	0.69
		70955	MA_118446_4316	T/A	Upstream variant	0.644	0.40
TWD	Slope	131698	MA_10235390_3386	G/A	Stop gained	0.672	1.58
		160208	MA_10433411_3386	T/C	Intron variant	0.595	3.41
	β_2_	89044	MA_212523_6278	T/C	Upstream variant	0.534	3.34
LWD	Slope	43797	MA_62987_13474	T/C	Missense variant	0.524	1.81
		165481	MA_10434805_21408	C/T	Intron variant	0.588	1.21
		171223	MA_10436058_4902	G/A	Intron variant	0.712	4.03
RW	Intercept	11535	MA_10694_9101	A/C	Synonymous variant	0.545	1.95
		112391	MA_879270_7373	C/T,A	Stop gained	0.532	1.45
		112394	MA_879384_3894	C/A	Splice region variant	0.692	2.56
	Slope	165481	MA_10434805_21408	C/T	Intron variant	0.521	2.66
	β_2_	23808	MA_20322_28351	T/G	Synonymous variant	0.554	1.78
		165481	MA_10434805_21408	C/T	Intron variant	0.533	0.18
	β_3_	23808	MA_20322_28351	T/G	Synonymous variant	0.55	1.20
		165481	MA_10434805_21408	C/T	Intron variant	0.615	1.79
TRW	Slope	111057	MA_817099_1105	T/A	Missense variant	0.685	1.12
	β_2_	33110	MA_38472_13803	T/A	Upstream gene variant	0.657	3.23
		89295	MA_214776_1624	G/A	Upstream gene variant	0.688	4.51
	β_3_	111057	MA_817099_1105	T/A	Missense variant	0.672	1.20
LRW	Intercept	143628	MA_10428744_29330	C/T	Downstream variant	0.668	0.5
	β_3_	164772	MA_10434624_20686	C/A	Downstream variant	0.571	0.06
MOE	Slope	165481	MA_10434805_21408	C/T	Intron variant	0.602	1.00
NC	β_2_	145839	MA_10429444_12692	G/C	Upstream variant	0.645	3.82
ENC	Slope	98508	MA_402880_2045	A/C	Upstream variant	0.667	0.03
		167610	MA_10435406_13733	A/G	Downstream variant	0.685	0.01
TNC	Intercept	95870	MA_346723_2241	T/C	Upstream variant	0.667	3.78
		126785	MA_9447489_687	A/C	Upstream gene variant	0.68	4.93
LNC	Intercept	143628	MA_10428744_29330	C/T	Downstream variant	0.66	3.14
	Slope	143628	MA_10428744_29330	C/T	Downstream variant	0.672	4.77
EP	Intercept	16868	MA_15729_40331	G/T	Intron variant	0.609	3.32
		91242	MA_246125_1213	G/A	Synonymous variant	0.594	3.41
TP	Intercept	101203	MA_462319_4322	A/C	Upstream gene variant	0.594	1.16
		132014	MA_10251995_2442	A/C	Upstream gene variant	0.601	3.22
LP	β_2_	162397	MA_10434007_77578	C/T	Upstream gene variant	0.892	1.14
EP/LP	Intercept	51657	MA_80954_29644	G/A	Downstream variant	0.63	0.81
		60787	MA_98424_947	C/T	Intron variant	0.655	1.80
		123639	MA_8790100_1384	A/C	Upstream variant	0.628	0.75
	β_2_	59480	MA_96191_7122	A/G	Synonymous	0.6	2.37
	β_3_	117333	MA_1045136_4310	T/C	Missense variant	0.523	1.34
Mass index (growth × density)	Intercept	166235	MA_10435002_4986	G/A	Intergenic variant	0.533	0.65
	Slope	61096	MA_99004_17108	G/A	Synonymous variant	0.66	0.01
		67181	MA_109804_10278	G/A	Missense variant	0.612	0.05
		1401	MA_1378_4718	C/A	Exon/stop gained	0.588	1.19
		138744	MA_10427214_13968	G/T	Missense variant	0.58	1.80
		162397	MA_10434007_77578	C/T	Upstream variant	0.627	1.44
	β_2_	21924	MA_19222_1789	A/G	Upstream variant	0.71	1.82

a SNP: The SNP name was composed of the contig (MA_number) and SNP position on contig. For example, the first SNP MA_1043540_13733 was located on contig MA_1043540 at position 13 733 bp; PVE is the phenotypic variance explained.

**Table 2:** SNP modes of inheritance

Phenotype	QTL	SNP	Allele	2a^a^	d^b^	d/a
WD	167610	MA_10435406_13733	A/G	19.63	9.45	0.96
	30469	MA_33109_11804	A/G	5.19	6.22	2.40
	157442	MA_10432646_63090	G/A	4.49	2.80	1.25
EWD	167610	MA_10435406_13733	A/G	5.495	2.270	0.83
	23798	MA_20321_44812	C/T	4.814	5.905	2.45
	70955	MA_118446_4316	T/A	1.989	0.966	0.97
TWD	131698	MA_10235390_3386	G/A	3.849	−0.636	−0.33
	160208	MA_10433411_3386	T/C	2.313	3.908	3.38
	89044	MA_212523_6278	T/C	0.703	−0.962	−2.73
LWD	43797	MA_62987_13474	T/C	5.124	−1.086	−0.42
	165481	MA_10434805_21408	C/T	4.684	1.165	0.50
	171223	MA_10436058_4902	G/A	0.938	2.482	5.29
RW	11535	MA_10694_9101	A/C	0.111	0.049	0.88
	112391	MA_879270_7373	C/T,A	0.056	−0.027	−0.98
	112394	MA_879384_3894	C/A	0.194	−0.045	−0.45
	165481	MA_10434805_21408	C/T	0.158	0.039	0.49
	23808	MA_20322_28351	T/G	0.025	0.030	2.62
TRW	111057	MA_817099_1105	T/A	0.016	0.001	0.16
	33110	MA_38472_13803	T/A	0.029	−0.002	−0.19
	89295	MA_214776_1624	G/A	0.026	−0.001	−0.13
LRW	143628	MA_10428744_29330	C/T	0.006	−0.002	−0.67
	164772	MA_10434624_20686	C/A	0.002	0.003	2.90
MOE	165481	MA_10434805_21408	C/T	0.376	0.101	0.53
NC	145839	MA_10429444_12692	G/C	0.298	0.792	5.31
ENC	98508	MA_402880_2045	A/C	4.144	1.314	0.63
	167610	MA_10435406_13733	A/G	4.695	−3.033	−1.29
TNC	95870	MA_346723_2241	T/C	0.529	−0.187	−0.71
	126785	MA_9447489_687	A/C	0.083	−0.429	−10.21
LNC	143628	MA_10428744_29330	C/T	0.219	−0.057	−0.52
EP	16868	MA_15729_40331	G/T	0.542	0.149	0.55
	91242	MA_246125_1213	G/A	0.183	−0.129	−1.40
TP	101203	MA_462319_4322	A/C	0.469	−0.199	−0.85
	132014	MA_10251995_2442	A/C	0.339	−0.429	−1.63
LP	162397	MA_10434007_77578	C/T	0.127	−0.071	−1.11
EP/LP	51657	MA_80954_29644	G/A	0.081	0.062	1.49
	60787	MA_98424_947	C/T	0.254	−0.181	−1.43
	123639	MA_8790100_1384	A/C	0.032	−0.078	−4.81
	59480	MA_96191_7122	A/G	0.120	−0.117	−1.95
	117333	MA_1045136_4310	T/C	0.018	0.077	8.56
	166235	MA_10435002_4986	G/A	0.138	0.013	0.19
MI	61096	MA_99004_17108	G/A	0.006	−0.009	−3.16
	67181	MA_109804_10278	G/A	0.007	−0.012	−3.14
	1401	MA_1378_4718	C/A	0.003	0.004	2.67
	138744	MA_10427214_13968	G/T	0.002	0.017	17.00
	162397	MA_10434007_77578	C/T	0.025	−0.010	−0.79
	21924	MA_19222_1789	A/G	0.014	−0.008	−1.14

a Calculated as the difference between the phenotype means observed within each homozygous class (2a = |G_BB_ − G_bb_|, where G_ij_ is the trait mean in the *ij*th genotype class).

b Calculated as the difference between the phenotypic mean observed within the heterozygous class and the average phenotypic mean across both homozygous classes [d = G_Bb −_ 0.5(G_BB_+G_bb_)], where G*ij* is the trait mean in the *ij*th genotypic class.

Previous work utilizing a functional mapping analysis in forest trees have used a limited number of molecular markers (Li *et al*., 2014). Li *et al*. (2014) applied this analysis in a bi‐parental Scots pine cross using 319 markers. Hence, our work represents an advance in that we have been able to apply this approach at the genome‐wide scale (178 101 SNPs) on maternal trees, with a dynamic trait dataset comprising 14 time points/annual growth rings (i.e. cambial age). Latent traits represent significant time points in the trait development allowing us to detect putative genes at these critical junctures in wood formation. Functional mapping has also been applied in ecological studies (Paine *et al*., 2012) and crops more recently. The fitting of growth models to the data describing growth trajectories of wood formation phenotypes allowed the identification of marker‐trait associations. This enabled us to track phenotype development against the genetic contributions at key time points.

Wood properties have previously been indicated to have a complex genetic architecture, in which association studies that make use of historical recombination represent a method that presents a substantial increase in QTL detection power for such complex traits (Hall *et al*., 2016). In our study, the number of QTLs detected reflected the complex nature of the traits under study, and our experimental design allowed the detection of the largest/most significant QTLs. A previous functional mapping study involving SNPs in conifers applied two levels of evaluating QTLs (Li *et al*., 2014), for which they have suggestive and significant QTLs, with our study only reporting the significant QTLs (single level), hence the small number of QTLs in our study. The small number of significant QTLs might also be due to the complex nature of the approximately 20 Gbp spruce genome. The sequence capture method only covered a total of 2331.1 kbp of exonic sequence, 2470.9 kbp of intronic sequence, 40.7 kbp of UTR‐like sequence and 9119 exon−intron boundaries (Vidalis *et al*., 2018). Therefore, a large portion of the genome was not represented and this would be compounded by the rapid LD in spruce, which might affect the number of significant QTLs detected. However, the numbers of QTLs detected in our study are in line with some previous studies in conifers (González‐Martínez *et al*., 2007), and with the drought association study in Norway spruce (29 significant SNP) (Trujillo‐Moya *et al*., 2018).

The QTL detected in our study explain a small proportion of the genetic variation and this could be due to several factors. This is in line with previous studies examining genetic variation in complex traits in coniferous species using forward genetic approaches, such as QTL (Sewell *et al*., 2000; Novaes *et al*., 2009) and AM (Wegrzyn *et al*., 2010; Du *et al*., 2013, 2018; Porth *et al*., 2013; McKown *et al*., 2014; Lamara *et al*., 2016) The large effective population size in forest tree populations closely resembles humans, therefore making the ‘missing heritability’ issue found in human AM experiments relevant to forest tree populations. First, one of the hypothesis attributed to this ‘missing heritability’ is the substantial amount of quantitative variation linked to the cumulative effect of rare alleles that cannot be detected in GWAS using small sample sizes. Therefore in our study increasing the sample size from 517 individuals might allow the inclusion of rare alleles, explaining some of the missing heritability (Hamblin *et al*., 2011; De La Torre *et al*., 2019). The detection of true low‐frequency alleles associated with complex traits is challenging as it requires large and genetically diverse populations (Hall *et al*., 2016). Variants with low minor allele frequencies are usually discarded due to the potential of genotyping errors. However, rare alleles play an important role in both the genetic regulation of traits and explaining the ‘missing heritability’ in forest species (De La Torre *et al*., 2019). Therefore, this could have also contributed to the small effect sizes detected in our study as we filtered SNPs with low minor allele frequencies (<0.05 MAF). Second, allelic heterogeneity in which multiple functional alleles exist and are associated with different phenotypes, especially for such complex traits as those linked with wood formation. The presence of allelic heterogeneity will require a large population size that will encompass the allelic variations to account for the missing heritability (Bergelson and Roux, 2010). Third, non‐additive effects mainly epistatically derived variation between genes might go undiscovered (Storey *et al*., 2005). Most GWAS models have been designed to only consider the additive effects of markers. Numerous studies have shown that non‐additive effects constitute a large part of the genetic variation of complex traits, these studies considered intra‐locus (dominance) and inter‐locus (epistatic) effects (Huang *et al*., 2012; Zhou *et al*., 2012; Mackay, 2013; Yang *et al*., 2014; Du *et al*., 2015). Yang *et al*. (2014) showed in corn an increase in the proportion of heritability explained when a model considering dominance was utilized and therefore allowing a better overview of heterosis. In rice Zhou *et al*. (2012) demonstrated the accumulation of multiple effects, including dominance and overdominance, which might partially explain some of the genetic basis for heterosis. Du *et al*. (2015) identified additive, dominant and epistatic effects explaining nearly two‐fold high heritability in *Populus tomentosa* for 10 growth and wood property traits utilizing pathway‐based multiple gene associations.

Lastly, epigenetic variation is also likely to be one of the sources of the ‘missing heritability’. With the development of advanced sequencing platforms, sophisticated genotyping tools have been developed to unravel epigenetic variation (Johannes *et al*., 2009). Therefore, the influence of each of these factors on heritability strongly depends on the population sampled and inclusion of sophisticated genotyping tools in the case of epigenetics. The incorporation of a combination of advanced statistical models such as regional heritability mapping (RHM) and the detection of structural variants, insertion/deletions (InDels) and copy number variants in GWAS studies from several tree species has resulted in higher heritabilities being detected (Resende *et al*., 2017a; Gong *et al*., 2018)

### Trait trajectories and functional mapping

**Figure 1:**
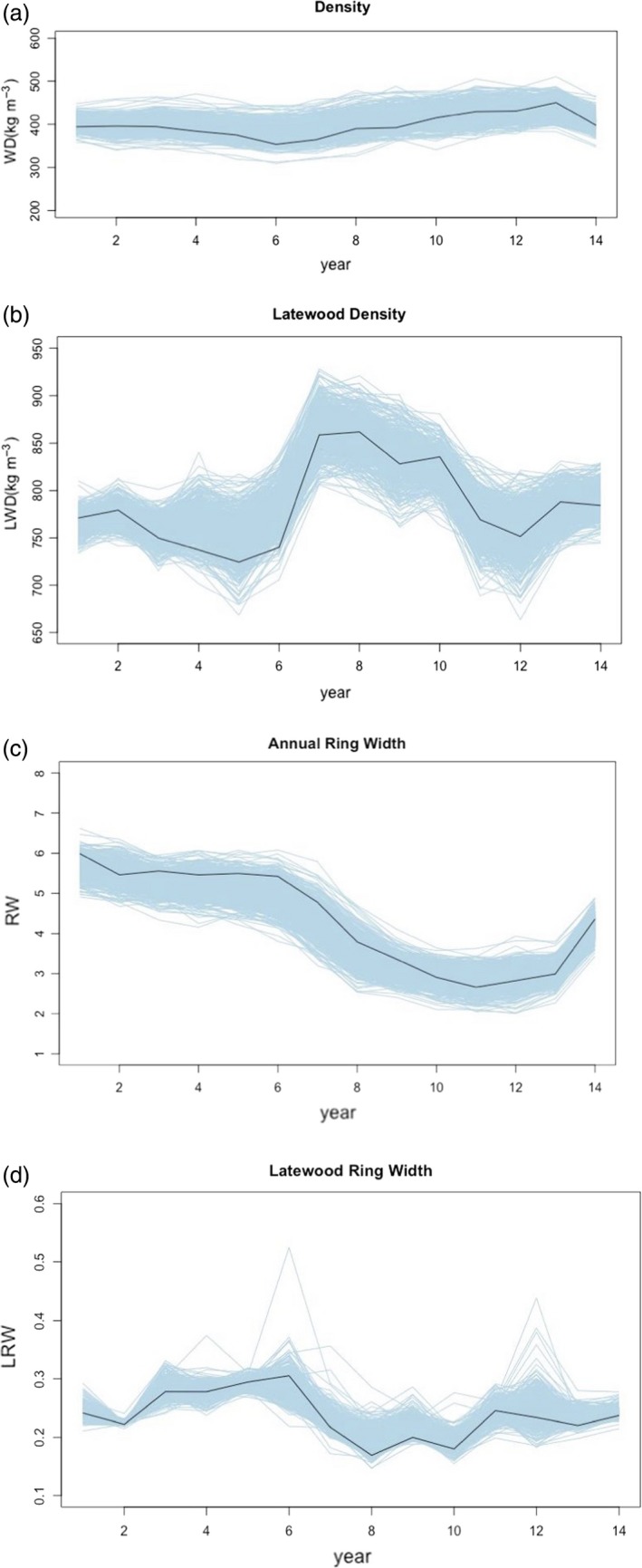
EBV trajectories of four wood quality traits over time: (a) wood density, (b) late wood density, (c) annual ring width and (d) late wood ring width. Individual trajectories for each trait are shown in light blue lines and the black line represents the mean trajectory for the phenotype. These trajectories were used to determine the four latent traits of each tree, using linear splines with two knots.

EBVs were plotted as phenotype data versus 14 consecutive cambial ages (Figure 1). All phenotypes under investigation are represented with thin light blue (Black average) curves, to visualize the nature of variation and growth trajectories of the phenotype (Figure 1 and Supporting Information Figure S1). The dissection of dynamic traits in forest trees has been predominantly performed using single data points representing the value of the trait at a given developmental stage. The major disadvantage of such an approach is that it overlooks many of the factors that define the process of formation and development for important traits such as density and ring width. We utilized splines that have the advantage of not making *a priori* assumption about the shape of the curve and allow for the trait growth trends to be unbiased. Splines also allow for the characterization of the dynamic traits in terms of a few parameters derived from the spline models (Al‐Tamimi *et al*., 2016). The fitting of growth trajectories is considered as optimal because it treats phenotypes measured over time as different traits and also takes into account the correlation generated by the ordered time points (Yang *et al*., 2006). The growth trajectories of the traits over time were calculated from the fitted splines and time intervals were identified and selected based on the characteristic growth trajectory of each trait, resulting in associations *across* and *within* traits being identified (Table 1). Therefore, indicating the control by different sets of genes at different time points for our longitudinal traits (Table 2), just as in some age‐specific QTLs found in other conifers and rice (Verhaegen *et al*., 1997; Emebiri *et al*., 1998; Wu *et al*., 1999). This approach has the potential to be applied to genomic prediction and selection studies for predicting individuals that would have the highest impact through the formation and development of a trait of interest. With application of differentially penalized regression (DiPR), pooled significant association markers can be utilized in GS in order to increase prediction accuracies (Bentley *et al*., 2014).

### Linkage disequilibrium

**Figure 2:**
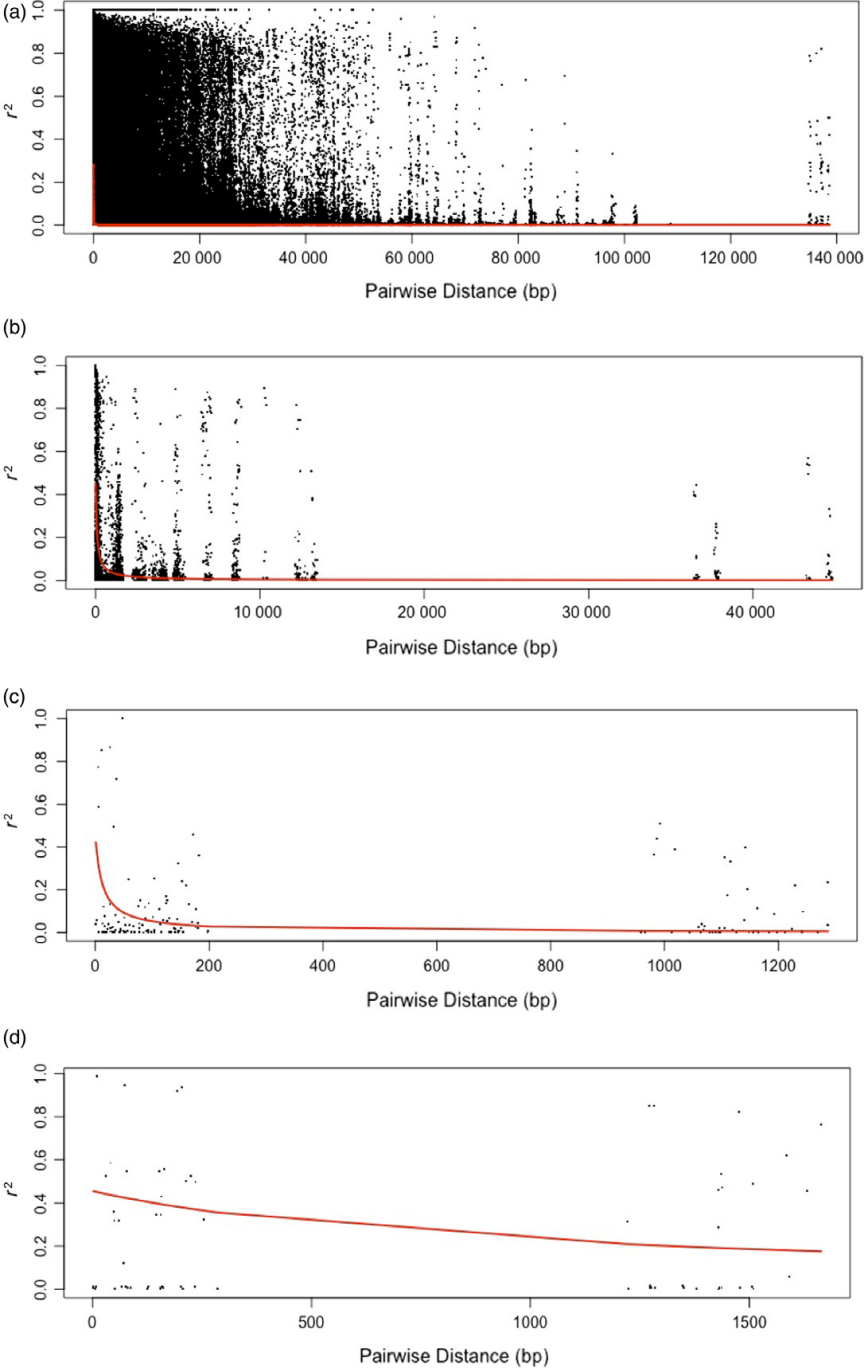
(a) Decay of linkage disequilibrium (LD) across all the tagged genomic sequences, the majority being exonic regions. The squared correlation coefficient between loci (r^2^) is plotted against distance, in base pairs, separating loci. The fitted curve (red) is representative of the trend of decay from the 178 101 SNPs utilized in the association mapping (AM). (b) Decay of LD with distance in base pairs between sites from across 41 contigs with significant associations. (c) Decay of LD across contig MA_96191 that has a significant association for ratio of percentage earlywood vs latewood on which two probes were captured. (d) Decay of LD on contig MA_80033 indicating the variable LD in the genome.

The zygotic LD (squared correlation coefficient *r* ^*2*^) was determined through the pooling of all *r* ^*2*^ values and plotting them against the physical distances between the same SNP pair (Figure 2a). This allowed us to estimate the genome‐wide degree of LD in Norway spruce, with the average LD for linked SNPs being inferred from the trendline (curve) of the nonlinear regressions. The fitted curve indicates the LD is low in Norway spruce, rapidly decaying by over 50% (from 0.50 to 0.20) (Figure 2a). The average distance associated with the LD decline for *r* ^*2*^ = 0.1 varied from 14 to 1500 bp (Figures 2c,d and S2). Neale and Savolainen (2004) reported an LD decayed to less than 0.20 within roughly 1500 bp based on 19 candidate genes in loblolly pine. As conifers are highly outcrossing a rapid LD decay is expected, however in spruces the LD displays diverse patterns among different genes or the same genes in different species. The LD decline in spruces was also noted to be roughly between a few base pairs and 2000 bp (Namroud *et al*., 2010). These diverse heterogenous LD patterns were also observed when we analyzed the LD for individual contigs that had significant associations to our traits (Figure 2c,d, Figure S3). The general LD estimate of all the SNP pairs indicated a fast LD decay (Figure 2a). This rapid decay could be due to the number of contigs analyzed in relation to the large Norway spruce genome, as well as the use of zygotic LD between genotypes. Lu *et al*. (2016), noted that the calculation of gametic LD from phased haplotypes indicated a slower LD decay than when using zygotic LD in loblolly pine. However, they also observed varying rates of LD decay between genes and across different genome regions (Lu *et al*., 2016). Therefore, the generality of the LD patterns within the Norway spruce genome remains to be further analyzed because only a relatively small and highly specific portions of the genome was studied here.

### Population inference

To account for effects derived from population stratification we performed a principal component analysis (PCA). The top two explained a total of 5.3% of the variation. Population structure inference of clusters detected by PCA was performed by ADMIXTURE (Figure S3) and the best K value plotted from the cross‐validation error term. Using the best K method, K = 2 better explained the genetic structure of the study population (Figure S3).

### Overall summary of genetic associations

Several associations were shared *within* each trait and *across* traits in the analysis. WD, Ring width (RW), Transitional ring witdh (TRW) and Latewood number of cells (LNC) had one (MA_33109_11804), two (MA_10434805_21408 and MA_20322_28351), one (MA_817099_1105) and one (MA_10428744_29330) QTLs shared by two or more latent traits, respectively. Common QTLs *within* RW were observed for slope*,* β_2_ and β_3_ latent traits, with moderate frequencies ranging from 0.521 to 0.615 and influenced their respective traits to modest degrees (PVE in ranges of 0.18–2.66%).

For QTLs common *across* the different latent traits, SNP MA_10434805_21408 was shared between latewood wood density (LWD), RW and Modulus of elasticity (MOE); this is not surprising because of the close correlation between MOE and wood density. Intron variant MA_10434805_21408 explained between 0.18 and 2.66% of the PVE observed in the respective traits. This SNP associated also had high frequencies of 0.602 and 0.615 in MOE and RW explaining PVE of 1.00 and 2.66%, respectively (Table 2). SNP MA_10435406_13733 was shared between WD, Earlywood wood density (EWD) and Earlywood number of cells (ENC), was associated with the intercept trait for WD and EWD and the slope latent trait in ENC (Table 1), with PVE ranging from 0.01 to 4.64%. The QTL had a high influence on the density related traits as it explained 4.64% (WD) and 3.38% (EWD) both exhibiting a partial dominant inheritance pattern (Table 2).

Numbers of cells (NC), ENC, TNC and LNC traits were associated with a total of three putative genes and three protein domains. Of the three putative genes, two are associated with serine/kinase activity and one is involved in cysteine and methionine synthesis (Table S1). All the SNPs associated with these traits were either downstream or upstream of coding regions and may therefore act as modifiers of gene expression.

Wood percentage traits, early wood proportion (EP), LP, TP and the ratio of EP/LP had significant associations with 10 SNPs. Four of the six significant SNP variants for EP/LP are modifiers with the other two SNPs, being a synonymous (MA_96191_7122) and missense (MA_1045136_4310) variant. The synonymous SNP MA_96191_7122 was consistent with the codominant mode of inheritance (Table 2). The significant SNP MA_15729_40331, an intron variant, that is associated with EP, is located in the gene MA_15729g0010, homologous to a DNA‐3‐methyladenine glycosylase II enzyme (Table 1).

WD, EWD, TWD and LWD had a total of 12 significant associations. A missense SNP, MA_33109_11804, was associated with WD and located within the gene homologous to an Arabidopsis senescence‐associated gene 24 (Table S1). Of the three significant SNP associations for Transitional wood density (TWD), two, SNP MA_10235390_3386 (stop gained) and SNP MA_212523_89044 (upstream gene variant) were identified within genes. Two of the three significant SNPs identified for LWD were intron variants (MA_10434805_21408 and MA_10436058_4902) with the third being a missense variant (MA_62987_13474).

**Figure 3:**
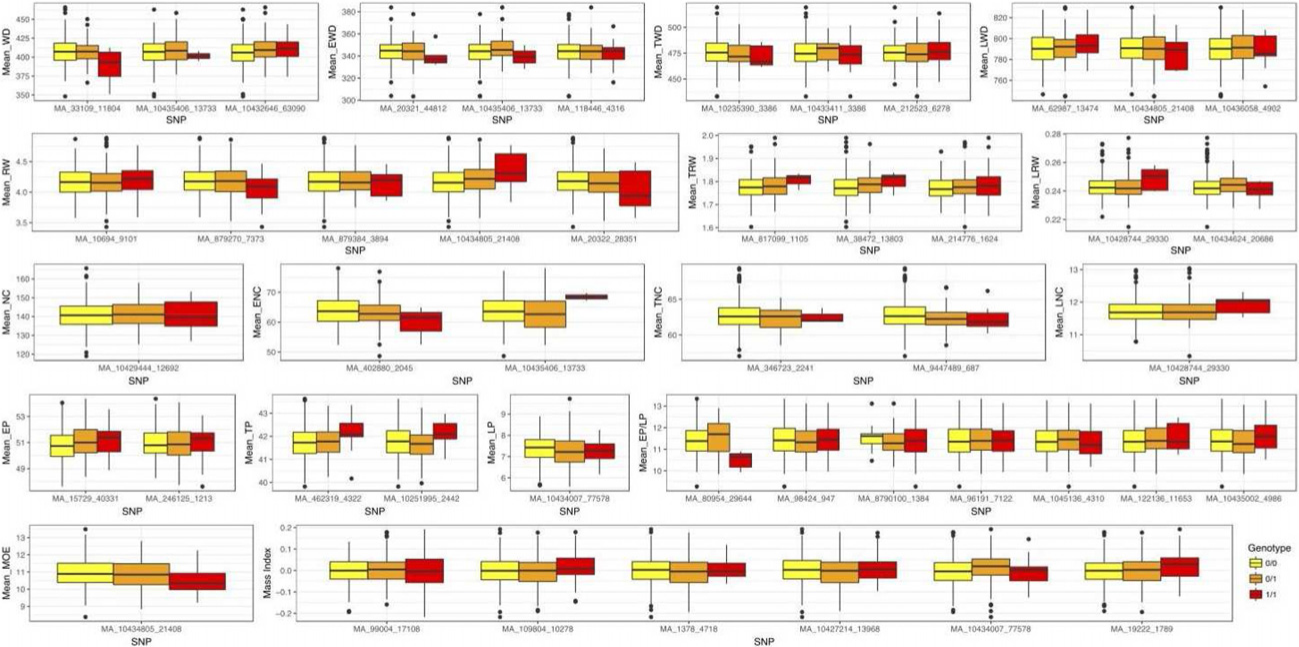
Box plot of the estimated genotypic effect on the phenotypes in the study. The significant SNPs associated and each one of the traits have been correlated to give the impact each genotype has on the average of the overall trait

Trees showing a positive correlation between growth and density had seven QTL specific for this observed phenomenon (MI), explaining a PVE ranging between 0.05 and 1.82% (Table 1). The seven associated SNPs, were two upstream gene variants, two missense variants one intergenic variant, one stop gained variant and one synonymous nucleotide replacement (Table 1). The SNP MA_1378g0010_4718 encodes for a premature stop codon on gene MA_1378g0010. Two SNPs associated with the slope latent trait for MI (MA_1378_4718 and MA_10427214_13968) have an overdominance inheritance pattern with the C and G alleles being dominant, respectively (Table 2; Figure 3).

### Genetics associations for genes of known function in wood formation

#### Intercept associations

Our study identified several interesting genes linked to the significant QTLs from the intercept latent trait, which represents the mean from our spline model. This resulted in 17 significant associations with a PVE ranging from 0.50 to 4.64% associated with the intercept latent trait. The modes of action determined by the non‐additive effects of these significant SNP associations to the intercept latent trait were one for overdominance (|*d/a*| > 1.25), codominant (|*d/a*| < 0.50) 12 and four SNPs were partial to fully dominant (0.50 < |*d/a*| < 1.25).

**Figure 4:**
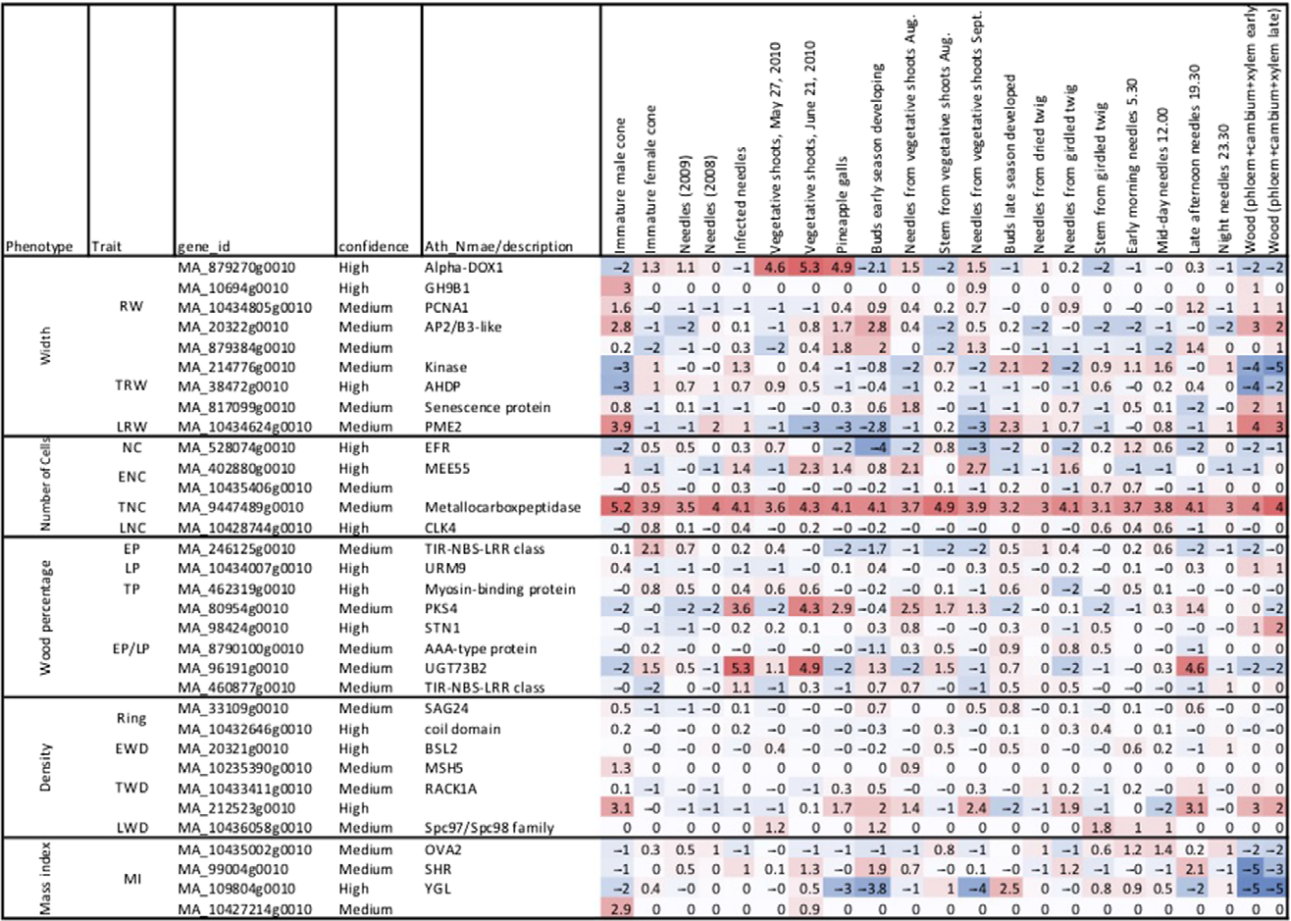
The heatmap showing the expression levels (VST values) of spruce candidate genes in different organs and tissues based on data of Nystedt *et al*. (2013) available at http://congenie.org.

Ring width phenotypes RW, TRW, and LRW were linked with a total of three gene models associated with the intercept latent trait (Table S1). Of these putative genes associated with RW phenotypes, gene MA_10694g0010 was of particular interest with regards to wood formation. SNP MA_10694_9101 with a partial to fully dominant mode of inheritance (Table 2) was located on the gene MA_10694g0010 that is homologous to an enzyme involved in cell wall biosynthesis, endoglucanase 11‐like, and was associated with RW (Table S1) and was expressed in the wood (phloem+cambium+xylem) component of spruce (Figure 4). This enzyme is a vital component of xylogenesis and is involved in the active digestion of the primary cell wall (Goulao *et al*., 2011). Endoglucanases have been proposed as enzymes involved in controlling cell wall loosening (Cosgrove, 2005). Endoglucanase 11‐like gene is part of the endo‐1 family in which the eno‐1‐4‐β‐glucanase *Korrigan* gene belongs. Characterization of the *Korrigan* gene in *P. glauca* has identified it to be functionally conserved and essential for cellulose synthesis (Maloney *et al*., 2012).

Density‐related phenotypes (WD, EWD, TWD and LWD) had two significant associations detected for the intercept. Both associations were detected by SNP MA_10435406_13733 and were both partial to fully dominant in their form of inheritance (Table 2). The SNP MA_10435406_13733 downstream on gene MA_10435406g0010 was also significantly associated with the trait ENC slope latent trait. The association of this gene with the WD and EWD intercept implies that it has an impact on the overall development of density throughout the growth period. This result coincides with previous report about the influence of the earlywood component on the properties of the annual ring as a whole in Scots pine (Li *et al*., 2014). Association of the same gene also with the slope latent trait of ENC corroborates the predictive value of number of cells for wood density. The gene is homologous to phosphoadenosine phosphosulfate reductase (PAPS), which plays a central role in the reduction of sulfur in plants. An analysis of PAPS enzymes in Arabidopsis (Klein and Papenbrock, 2004) and *Populus* (Kopriva *et al*., 2004) revealed that enzymes involved in sulfate conjugation play an important role in plant growth and development (Klein and Papenbrock, 2004).

#### Slope associations

The analysis of slope (rate of change) of wood formation traits over cambial ages, to our knowledge, has never been dissected in any Norway spruce QTL or AM analyses.

For slope latent traits two significant candidate genes concerning wood formation, PAPS and Proliferating Cell Nuclear Antigen (PCNA), were detected *across* related traits density, growth number of cells and MOE. The codominant SNP MA_10435406_13733 (Table 2) that is a 3′‐gene variant for WD was located on a gene that is homologous to a phosphoadenosine phosphosulfate reductase gene cysH_2 and common *across* ENC, WD and EWD.

The SNP MA_10434805_21408 was located on the gene MA_10434805g0010, which is homologous to an Arabidopsis PCNA protein (Table S1) and was ubiquitously expressed with high levels in the wood (phloem+cambium+xylem) component of spruce (Figure 4). This SNP is associated *across* LWD, RW and MOE with partial to fully dominance (0.50 < |*d/a*| < 1.25) for all three associations (Table 2). The presence of these common QTL suggests that these traits might be under the control of the same genes or genetic pathways. Chen *et al*. (2014) reported a significant positive genetic correlation between wood density and MOE, which increased with tree age. However, wood volume growth and density have a negative correlation (Chen *et al*., 2014), our study was able to detect QTLs for trees exhibiting a positive correlation for this phenomenon (MI). The common QTL observed *across* WD, EWD and ENC indicates that the number of cells during the juvenile wood development stages has a significant impact on the overall density. The seasonal changes in EWD to LWD have been speculated to be due to a change in auxin levels leading to the initiation of wall‐thickening phase, which has a direct impact on the wood quality traits such as MOE. This phase coincides with the cessation of height growth and where available resources are used for cell wall thickening (Sewell *et al*., 2000), which may explain the common QTL between LWD, RW and MOE, as part of the same feedback loop mechanism.

#### β_2_ and β_3_

When analyzing QTLs associated with the two latent traits β_2_ and β_3_, 16 significant associations were detected, with phenotypic variances ranging from 0.01 to 4.51% (Table 1). Five of the significant markers were consistent with overdominance (|*d/a*| > 1.25), with the 11 markers being dominant (0.50 < |*d/a*| < 1.25) (Table 2, Figure 3).

Wood density phenotypes (WD, EWD, TWD and LWD) had three significant associations with the β_2_ and β_3_ latent traits (Table 1). The upstream variant MA_33109_11804 associated with both the slope and β_2_ latent traits of WD was detected on the gene model MA_33109g0100 that is homologous to the *Arabidopsis* senescence‐associated gene 24 (Table S1) and the gene being expressed in shoots and buds of spruce (Figure 4). An association for the latent trait β_2_ of TWD with a codominant SNP (MA_212523_6278) (Table 2) was located upstream of gene MA_212523g0010 homologous to Kinesin‐related protein 13 (gene‐L484_021891) and ubiquitously expressed in shoot, buds and wood component of spruce, indicating its important function in this species (Figure 4). This association is of interest because Kinesin‐related proteins are known to be involved in secondary wall deposition, which can impact wood density (Zhong *et al*., 2002), cell wall strength and oriented deposition of cellulose microfibrils. Therefore, these proteins would have a direct correlation with the increase in density at the latent trait β_2_ at age 6 years (Figure S1).

Ring width phenotypes (RW, TRW and LRW) had eight significant associations identified for the latent traits β_2_ and β_3_, explaining PVE ranging from 0.01 to 4.51% (Table 1). The synonymous SNP MA_2032_28351 associated with the β_2_ latent trait for RW is located on a gene homologous with a plant‐specific B3‐DNA binding protein domain explaining 1.78% variation and is shared among various plant‐specific transcription factors. This includes transcription factors involved in auxin and abscisic acid responsive transcription (Yamasaki *et al*., 2004). Auxin is one of the central phytohormones in the control of plant growth and development (Abel and Theologis, 1996), and also known to be involved in cell wall loosening and elongation (Cosgrove, 2016). This association was detected *within* the RW phenotype and detected for both β_2_ and β_3_ latent traits (Table 1). Therefore, this domain could be involved with transcription factors involved in both the decrease and increase of RW (Figure S1). Three putative genes were associated with the β_2_ and β_3_ latent traits for the TRW phenotype. Of interest a senescence‐associated protein associated on the TRW β_2_ latent trait with the missense variant MA_817099_1105. This might be linked to the decrease in TRW at year 6 (Figure S1) due to the decline of photosynthetic rate known to be induced by the activity of senescence related proteins (Sillanpää *et al*., 2005). The gene was highly expressed in both the early and late wood components of spruce supporting the row of these senescence genes in controlling tree growth (Figure 4). This association was also identified for the slope latent trait indicating a potential impact on the rate of change of transitional wood. The detection of senescence related genes for wood density related phenotypes for both the slope and β_2_ latent trait (MA_33109_11804) could indicate a possible relationship between the genes influencing RW and wood density. Contig MA_10434624 is homologous to a pectin esterase and was associated with the downstream variant MA_10434624_20686 with an over‐dominant mode of inheritance for LRW (β_3_ latent trait) at year 10 (Table 2, Figure S1). This significant downstream SNP (MA_10434624_20686) associated with LRW on gene MA_10434624g0020 and homologous to pectin methylesterases (PMEs), which are cell wall associated enzymes responsible for demethylation of polygalacturonans (Phan *et al*., 2007). This gene was observed highly expressed in developing wood (Figure 4), indicating its importance for growth in spruce. This enzyme has been shown to be linked with many developmental processes in plants, such as, cellular adhesion and stem elongation (Micheli, 2001). An association study in *Picea glauca* (Moench) Voss identified a significant nonsynonymous SNP coding for cysteine associated with earlywood and total wood cell wall thickness associated with pectin methylesterase (Beaulieu *et al*., 2011). Our study identified a PME SNP association in the late wood stage, supporting the importance of PMEs in wood cell development.

When analyzing QTLs detected for traits linked to the percentage of cells (EP, LP and EP/LP) we identified three putative candidate genes, DNA‐3‐methyladenine glycosylase II enzyme, phytochrome kinase substrate 1 and glycosyltransferase. Synonymous SNP (MA_96191_59480) within the gene MA_96191g0010, which is homologous to Glucosyltransferase in *Picea sitchensis* was associated with the EP/LP, β_2_ latent trait. The gene is highly expressed in vegetative shoots (June) and during the late afternoon in needles (Figure 4). Glycosyl transferases operate by facilitating the catalytic sequential transfer of sugars from activated donors to acceptor molecules that form region and stereospecific glycosidic linkages (Lairson *et al*., 2008). The Arabidopsis ortholog (UDP‐glucosyltransferase 73B2) encodes for a putative flavonol 7‐*O*‐glucosyltransferase involved in stress responses. In our study, this significant association was associated with EP/LP, however a nonsynonymous variant in a gene coding for a glycosyl transferase in *Populus* was associated with fibre development and elongation (Porth *et al*., 2013).

Several receptor‐like kinases (TIR/NBS/LRR and serine/threonine‐protein phosphatase) homologues were identified *across* traits (TRW, NC, EP, EP/LP and EWD) (Table S1). Approximately 2.5% of the annotated genes in Arabidopsis genome are RLK homologues (Shiu and Bleecker, 2001), in which they, among other functions, play an important role in the differentiation and separation of xylem and phloem cells (Fisher and Turner, 2007). Similar to our study a synonymous SNP in an RLK gene was associated with EP in white spruce (Beaulieu *et al*., 2011), hence RLKs seem to be involved in modifying a number of different wood properties from density to cell identity and number.

Norway spruce trees that possess the ability of fast growth and high wood density are very rare, but such trees and associated SNPs were discovered in our study. Trees combining these traits are of high interest to the forestry industry. Of the seven genes significantly linked to this phenomenon of particular interest was a synonymous SNP on MA_99004g0100 gene homologous to a transcription factor from the GRAS family (Table S1). GRAS is an important class of plant‐specific proteins derived from three members: GIBBERELLIC ACID INSENSITIVE (GAI), REPRESSOR of GAI (RGA) and SCARECROW (SCR) (GRAS) (Hirsch and Oldroyd, 2009). GRAS genes are known to be involved in the regulation of plant development through the regulation of gibberellic acid (GA) and light signalling (Hirsch and Oldroyd, 2009; Cenci and Rouard, 2017). Furthermore GA signalling has also been shown to stimulate wood formation in *Populus* (Mauriat and Moritz, 2009). Therefore, the GRAS transcription factor identified here and the other six genes positively associated with MI provide interesting genetic markers and tools to understand this phenomenon.

Wood density traits were associated with a total of 12 genes, the largest number of genes identified from the contigs. The percentage of wood was linked to 10 putative genes, cell width had nine putative genes and number of cells was associated with six genes. Two genes were shared *across* multiple traits, PCNA was common *across* RW and LWD, and phosphoadenosine phosphosulfate reductase was shared *across* WD, EWD and ENC.

## Experimental procedures

### Plant material and phenotype data

Plant material and phenotype data used in this study have previously been described in Chen *et al*. (2014). In brief, two progeny trials were established in 1990 in southern Sweden (S21F9021146 aka F1146 (trial1) and S21F9021147 aka F1147 (trial2)). We selected 517 families originating from 112 sampling stands to use in the investigation of wood properties. At each site, increment wood cores of 12 mm were collected at breast height from six trees of the selected families (1.3 m) (6 progeny × 2 sites = 12 progenies in total). In total, 5618 trees, 2973 and 2645 trees from the F1146 and F1147 trials respectively, were analysed. The pith to bark profiling of growth and wood physical attributes was performed using the SilviScan instrument (Evans and Ilic, 2001) at Innventia, now part of RISE, Stockholm, Sweden, where also the initial data evaluations were performed (Methods S2). These included the identification and dating of all annual rings and their compartments of early wood (EW), transition wood (TW) and late wood (LW). For this, a density‐based ‘20‐80’ definition was used, described and discussed in (Lundqvist *et al*., 2018). Traits of interest to breeders were derived from the SilviScan data, such as the radial NC and Mass Index (MI) introduced to express the relative amount of biomass at breast height.

The investigation was trigged by the observation that some trees broke the unfavourable negative correlation of the trait MI which is between density and growth. They produced, more biomass than expected, and it was therefore important. In order to identify putative genes behind high values for this trait. MI was defined as:

$$\begin{array}{cc} \mathrm{Massindex} & =\left(\mathrm{Individual}\mathrm{cross}-\mathrm{sectional}\mathrm{average}\mathrm{density}\right.\\ \left.\;/populationcross-sectionalaveragedensity\right)\\ {}*\left(\mathrm{individual}\mathrm{cross}-\mathrm{sectional}\mathrm{area}\right.\\ \left./\mathrm{population}\mathrm{average}\mathrm{cross}-\mathrm{sectional}\mathrm{area}\right) \end{array}$$

The traits investigated in this study are listed in Table 3.

**Table 3:** List of the phenotypes, their abbreviations and measurement unit

Phenotype	Abbreviation	Unit
Ring wood density	WD	kg m^−3^
Early wood density	EWD	kg m^−3^
Transition wood density	TWD	kg m^−3^
Late wood density	LWD	kg m^−3^
Ring width	RW	μm
Early wood ring width	ERW	μm
Transition wood ring width	TRW	μm
Late wood ring width	LRW	μm
Ring number of cells	NC	
Early wood number of cells	ENC	
Transition wood number of cells	TNC	
Late wood number of cells	LNC	
Early wood percentage	EP	%
Transition wood percentage	TP	%
Late wood percentage	LP	%
Early/late wood percentage	EP/LP	%
Modulus of elasticity	MOE	GPa
Mass index (density × growth)	MI	

### Statistical analysis

EBVs were calculated for growth and wood quality traits for 14 consecutive annual growth rings. The variance and covariance components were estimated using asreml 4.0 (Gilmour *et al*., 2014) as described in Chen *et al*. (2014). In brief, the EBVs at each cambial age were estimated using univariate, bivariate or multivariate mixed linear models. The following univariate linear mixed model for joint‐site analysis was fitted to calculate EBV:

(1) $$Y_{\mathrm{ijkl}}=\mu +S_{i}+B_{j(i)}+F_{k}+SF_{\mathrm{ik}}+e_{\mathrm{ijkl}}$$

where *Y*_*ijkl*_ is the observation on the *l*th tree from the *k*th family in *j*th block within the *i*th site, μ is the general mean, *S*_*i*_ and $B_{j\left(i\right)}$ are the fixed effects of the *i*th site and the *j*th block within the ith site, respectively, *F*_*k*_ and *SF*_*ik*_ are the random effects of the *k*th family and the random interactive effect of the *i*th site and *k*th family, respectively, *e*_*ijkl*_ is the random residual effect. The random family and site by family interaction effects are assumed to follow $N\left(0,\sigma_{f}^{2}\right)$ $N\left(0,\sigma_{\mathrm{sf}}^{2}\right)$ and, respectively, where $\sigma_{f}^{2}$ and $\sigma_{\mathrm{sf}}^{2}$ are the estimated family genetic variance and site by family interaction variance, respectively. Residual variation *e* was assumed to $N\left(0,\left[\begin{array}{c} I_{n1}\sigma_{e1}^{2}0\\ 0I_{n2}\sigma_{e2}^{2} \end{array}\right]\right)$, where $\sigma_{e_{1}}^{2}$ and $\sigma_{e_{2}}^{2}$ are the residual variances for site 1 and site 2, *I*_*n*1_ and *I*_*n*2_ are identity matrices, *n1* and *n2* are the number of individuals in each site. The fit of different models was evaluated using the Akaike Information Criteria (AIC) and the optimal model was selected based on a compromise of model fit and complexity.

### Latent traits

The EBVs were plotted against cambial age (annual ring number) to produce time trajectories for each trait (Figures 1 and S1). Spline model was fitted to the trajectories and their curve parameters describing the character of their development over time were used as latent traits in order to describe the dynamics of the EBVs across age.

The general definition of a linear spline with multiple knots is as follows

(2) $$y\left(t\right)=\beta_{0}+\beta_{1}t+\beta_{2}{\left(t-K_{1}\right)}_{+}+\beta_{3}{\left(t-K_{2}\right)}_{+}+\ldots +\beta_{1+m}{\left(t-K_{m}\right)}_{+},$$

which is continuous and where *K*_i_ (*i *=* *1, … ,*m*;* K*_1_ < *K*_2_ … <*K*_*m*_) are defined as knots, and (*t − K*_*i*_)_*+*_ *= *(*t − K*_*i*_) if *t > K*_*i*_ (*K*_*i*_ **> 0; *i *=* *1, … ,*m*), and otherwise is equal to zero. The number of knots has to be properly defined to provide an accurate description of the data under investigation, while avoiding overadaptation to data (Li and Sillanpää, 2015; Camargo *et al*., 2018). In our case, we found two knots most suitable to the time intervals investigated. Hence, the linear spline model to describe the growth trajectory of individual *i* applied in this study was defined as:

(3) $$y\left(t\right)=\beta_{0}+\beta_{1}t+\beta_{2}{\left(t-K_{1}\right)}_{+}+\beta_{3}{\left(t-K_{2}\right)}_{+}+\varepsilon_{i}\left(t\right),\;\varepsilon_{i}\left(t\right)\overset{\mathrm{iid}}{∼}N\left(0,\sigma^{2}\right).$$

In equation (2), the intercept β_0_, slope parameters β_1_, β_2_ (at Knot 1 (*K*_*1*_)) and β_3_ (at Knot 2 (*K*_*2*_)) are estimated by standard least squares (Ruppert *et al*., 2003). The four estimates were used as the latent trait in the subsequent QTL analysis conducted in rstudio (Team, 2015), and then analysed using the LASSO model to identify SNPs showing significant associations to the traits.

The intercept and slopes were used to evaluate the mean and rate of change for the trait across the annual rings, respectively. β_2_ and β_3_ represent inflection points in the cambial age trajectories where the development of the EBVs enters new phases. These two points (β_2_ and β_3_) are therefore supposed to have biological significance, warranting a closer analysis of the genes imparting these shifts in the EBVs dynamics. The four latent traits show lower correlations compared with the direct measurements on the original scales and they also have constant variances, thereby reducing the need to account for residual dependencies in the model (Wu *et al*., 2004; Yang and Xu, 2007; Li *et al*., 2014).

### Sequence capture, genotyping and SNP annotation

Total genomic DNA was extracted from 517 maternal trees, using the Qiagen Plant DNA extraction (Qiagen, Hilden, Germany) protocol with DNA quantification performed using the Qubit^®^ ds DNA Broad Range (BR) Assay Kit (Oregon, USA). Extracted DNA was submitted to RAPiD Genomics (USA) where DNA library preparation and capture sequencing were performed. Sequence capture was performed using the 40 018 diploid probes designed and evaluated for *P. abies* (Vidalis *et al*., 2018). The Illumina sequencing compatible libraries were amplified with 14 cycles of polymerase chain reaction (PCR) and the probes were then hybridized to a pool comprising 500 ng of 8 equimolar combined libraries following Agilent's SureSelect Target Enrichment System (Agilent Technologies, https://www.agilent.com/). These enriched libraries were then sequenced using an Illumina HiSeq 2500 instrument (San Diego, USA) on the 2 × 100 bp sequencing mode.

Raw reads were mapped against the *P. abies* reference genome v.1.0 using bwa‐mem (Li, 2013). samtools v.1.2 (Li *et al*., 2009) and Picard (http://broadinstitute.github.io/picard) were used for sorting and marking of PCR duplicates. Variant calling was performed using gatk haplotypecaller v.3.6 (Van der Auwera *et al*., 2013) in gVCF output format. Samples were then merged into batches of approximately 200 before all 517 samples were jointly called.

Variant Quality Score Recalibration (VQSR) method was performed to avoid the use of hard filtering for exome/sequence capture data. For the VQSR analysis two datasets were created, a training subset and an input file. The training dataset was derived from the Norway spruce genetic mapping population showing expected segregation patterns (Bernhardsson *et al*., 2019) and assigned a prior value of 15.0. The input file was derived from the raw sequence data using gatk with the following parameters: extended probe coordinates by +100 excluding INDELS, excluding LowQual sites, and keeping only bi‐allelic sites. The following annotation parameters QualByDepth (QD), MappingQuality (MQ) and BaseQRankSum, with tranches 100, 99.9, 99.0 and 90.0 were then applied for the determination of the good versus bad variant annotation profiles. After obtaining the variant annotation profiles, the recalibration was then applied to filter the raw variants. Using vcftools v.0.1.13 (Danecek *et al*., 2011), SNP trimming and cleaning involved the removal of any SNP with a MAF and ‘missingness’ of <0.05 and >20%, respectively. The resultant SNPs were annotated using default parameters for snpeff 4 (Cingolani *et al*., 2012). Ensembl general feature format (GFF; gene sets) information was utilized to build the *P. abies* snpeff database.

### Genetic structure and mode of inheritance

Linkage disequilibrium was calculated as the squared correlation coefficient between genotypes (*r* ^2^), globally with special attention given to all the contigs with significant associations in vcftools v.0.1.13 software using the ‘geno‐r2’ routine (Danecek *et al*., 2011). The trendline of LD decay with physical distance was fitted using nonlinear regression (Hill and Weir, 1988) and the regression line was displayed using rstudio (Team, 2015). Non‐additive effects of the significant markers was determined using the ratio of dominance (*d*) to additive (*a*). The ranges were: partial or complete dominance (−0.50 < |*d/a*| < 1.25) and additive (−0.50 ≤ |*d/a*| ≤ 0.50), with |*d/a*| > 1.25 being equal to over‐ or underdominance (Eckert *et al*., 2009).

FactoMiner (Multivariate Exploratory Data Analysis and Data Mining) (Husson *et al*., 2017) implemented in rstudio software was used to perform PCA. The covariate matrix derived from the PCA was then displayed by plotting principal component 1 scores against principal component 2 scores. The components of the PCA covariate matrix were then applied to the AM to account for population structure and correcting for any stratification within the study. Significance of each genetic principal component (PC) was determined using the Tracy‐Widom (TWi) distribution and a significance threshold of *P *=* *0.01. For population clustering, admixture v.1.3.0 (Alexander *et al*., 2009) was used with five‐fold cross‐validation and 200 bootstrap replicates. The bestK method was implemented in rstudio to determine the best K with the use of an elbow plot on the cross‐validation error.

### Trait association mapping

It is natural to use LASSO method for simultaneous estimation of SNP effects and selecting a sparse subset of trait‐associated SNPs to the multilocus association model. This is because LASSO has nice properties like being able to handle high‐dimensional cases with p>>n (*i.e*., a number of SNPs much larger than number of individuals) and selecting only a single representative SNP from the group of highly dependent SNPs. The LASSO model as described by Li *et al*. (2014), was applied to all latent traits for the detection of QTLs.

The LASSO model:

(4) $$\min_{\left(\alpha_{0}\;,\alpha_{j}\right)}\frac{1}{2n}\sum_{i=1}^{n}{\left(y_{i}-\alpha_{0}-\sum_{j=1}^{p}x_{\mathrm{ij}}\alpha_{j}\right)}^{2}+\lambda \sum_{j=1}^{p}|\alpha_{j}|,$$

where *y*_*i*_ is the phenotypic value of an individual *i* (*i *=* *1,…,*n*;* n* is the total number of individuals) for the latent trait β_0_, β_1_, β_2_ or β_3_, α_0_ is the population mean parameter, *x*_*ij*_ is the genotypic value of individual *i* and marker *j* coded as 0, 1 and 2 for three marker genotypes AA, AB and BB, respectively, α_*j*_ is the effect of marker *j* (*i *=* *1,…,*n*;* n* is the total number of markers), and λ (>0) is a shrinkage tuning parameter. The penalty term is able to shrink the additive effects of some of the markers exactly to zero, and select a subset of the most important markers into the model. The tuning parameter λ determines the degree of shrinkage, and the number of markers having non‐zero effects. Cross‐validation is used to decide an optimal value for λ.

In *stability selection* (Meinshausen and Bühlmann, 2010; Alexander and Lange, 2011): (i) several bootstrap samples are first created from the original data; and (ii) frequency over‐bootstrap samples on how many times each SNP is being selected to the LASSO model is monitored and used as a stable measure of variable selection. Stability selection probability (SSP) of each SNP being selected to the model was applied as a way to control the false discovery rate and determine significant SNPs (Gao *et al*., 2014; Li and Sillanpää, 2015). Briefly a subsample of half the number of individuals was randomly picked up and the LASSO was performed on it to select a set of markers. This procedure was repeated 1000 times. Then the selection frequency of each marker being selected was calculated, and was used to judge the support of QTL. A decision rule suggested by Meinshausen and Bühlmann (2010) was applied to control the expected number of false positives:

(5) $$\frac{1}{2}+\frac{q^{2}}{2E\left[V\right]p},$$

where *q* is the number of selected markers, *E*[*V*] is the expected number of false positives, and *p* is the total number of markers. For a marker to be declared as a significant QTL, a SSP inclusion frequency of at least 0.52 (i.e. derived based on formula 5) was used for all traits. This frequency was inferred conditional on the expected number of false selected markers being less than one (Bühlmann *et al*., 2014).

Population structure was accounted for in all analyses by including the first five PCs based on the genotype data as covariates into the model. An adaptive LASSO approach (Zou, 2006) was used to determine the percentage of phenotypic variance (PVE) (*H* ^2^_QT_) of all the QTLs (Methods S1). These analysis were all performed in rstudio (Team, 2015).

### Candidate gene mining

To assess putative functionality of SNPs with significant associations, a gene enrichment analysis of putative genes and their associated orthologs was performed against the norwood v1.0 database (http://norwood.congenie.org) hosted by congenie (http://congenie.org/). The complete *P. abies* contigs that harboured the QTLs that were not annotated in the congenie were used to perform a nucleotide blast (blastn) search, using the option for only highly similar sequences (megablast) in the National Center for Biotechnology Information (NCBI) nucleotide collection database (https://blast.ncbi.nlm.nih.gov/Blast.cgi?).

## Conclusion

This work has dissected the genetic basis of wood properties in Norway spruce with use of functional AM. In total, we identified 52 significant QTLs for wood properties and mining of candidate genes located in the vicinity of significant QTLs identified genes that could be directly or indirectly responsible for variations in the observed traits. Functional mapping analyses allowed us to utilize all the longitudinal data for a trait simultaneously and may better account for the temporal trends and correlation structures across years for the complex traits associated with wood formation. It can therefore be applied to the detection of QTLs stable over time (i.e. the QTLs associated with intercept traits) with greater statistical evidence. The slope latent trait over cambial ages or the rate of juvenile‐to‐mature wood transition has allowed for the dissection the dynamics of the transition process itself and can be applied to other important plant breeding traits. The significance of our results is provided by the identification of QTLs associated to both high wood density and fast growth, therefore larger biomass. These QTLs can now be a basis for future functional genomics in Norway spruce.

However, the direct use of QTLs for marker‐assisted breeding has not been successful, mainly due to the difficulty in transferring the associations across populations and species of forest trees. With the small percentage variances detected and no direct information about the developmental change of QTL expression, breeders will be unable to make use of these QTLs in direct early selection. Non‐additive interactions especially epistasis, play an important role in accounting for the total genetic variance of a trait. Therefore this study will be a good basis for initiating the detection and estimation of possible epistatic influence on these complex traits. Future work should focus on replicated sampling from a larger number of representative genotypes across different environments, which take into consideration genotype × environment interactions. Additional support for marker‐assisted tree breeding may also be provided by the functional genetics studies, systems mapping and consideration of biological mechanisms (Liu and Yan, 2019) of the identified candidate genes in model trees like *Populus* sp.

## Conflict of interest

The authors declare no competing interests.

## Supporting information


**Figure S1.** Phenotype trajectories representing the main traits.
**Figure S2.** Significant contigs LD heatmap.
**Figure S3.** ADMIXTURE plot of the entire population.
**Figure S4.** Data are structured into three categories.

**Table S1.** ConGenIE BLAST search of contigs with significant QTLs.
**Table S2.** Ring‐related data (B): list of variables and examples of data.
**Table S3.** Curve shape data (A): list of variables for each property and example of data.

**Methods S1.** PVE evaluation of a QTL.
**Methods S2.** Trait data set used for GWAS identification of novel candidate loci affecting number of tracheids formed, radial growth, density, stiffness and mass at breast height of young Norway spruce.

## Data Availability

All the latent traits, genotypic data, SNP position files the AM scripts used for the analysis are publicly available at zenodo.org at https://doi.org/10.5281/zenodo.1480536. Raw sequence data for all the samples utilized in the study are found through the European Nucleotide Archive under accession number PRJEB29652. The Norway spruce genome assemblies and resources are available from http://congenie.org/pabiesgenome.
