# Supplementary figures and images for: Genome‐wide association study identified novel candidate loci affecting wood formation in Norway spruce
Source: Plant J. 2019 Jul 28;100(1):83–100. doi: 10.1111/tpj.14429 (PMC6852177; doi:10.1111/tpj.14429)

Association mapping identified candidate loci affecting wood formation in Norway spruce


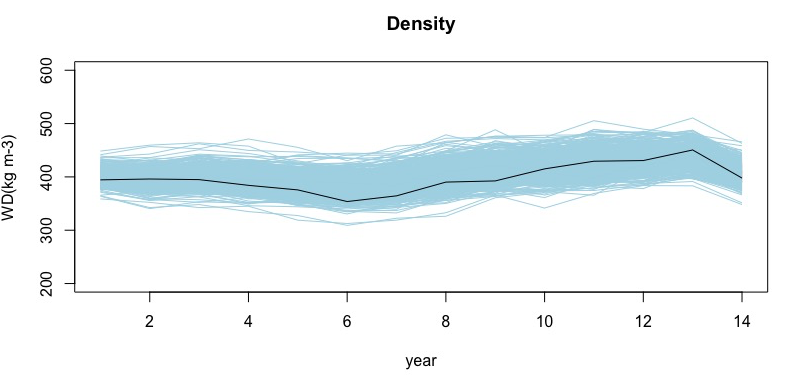


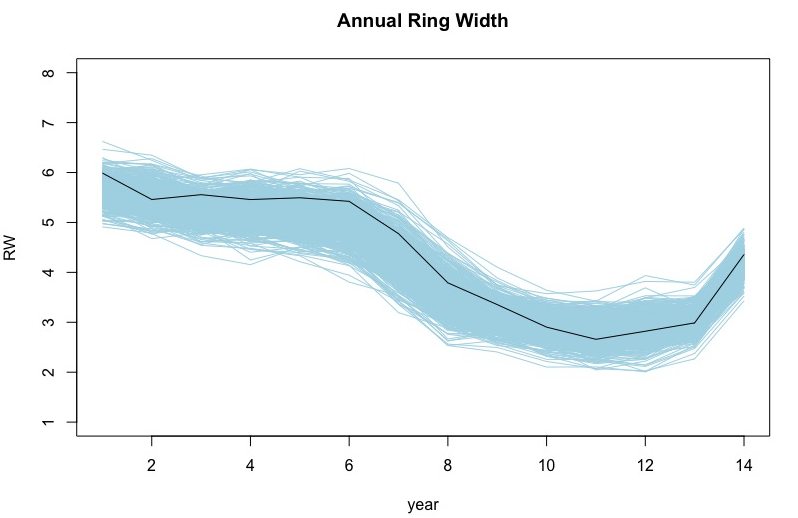


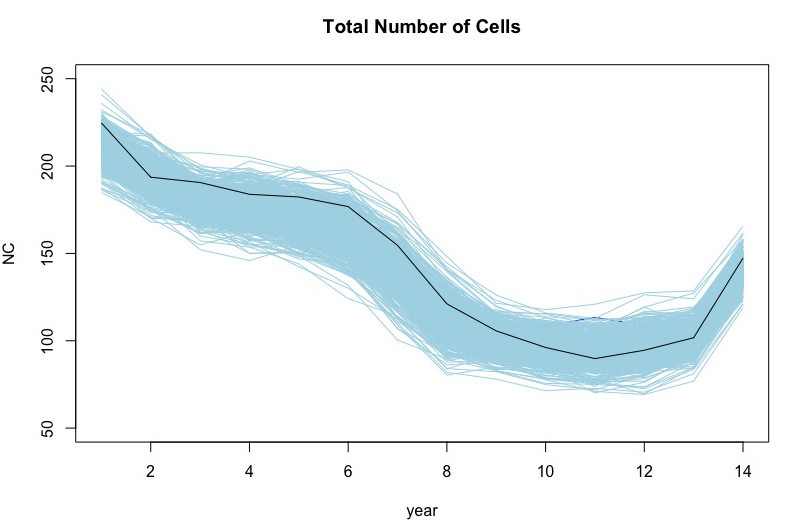


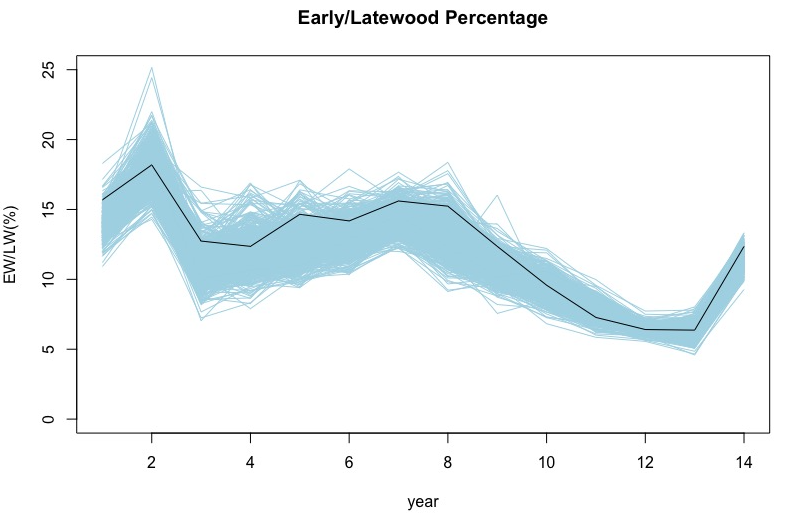


A)


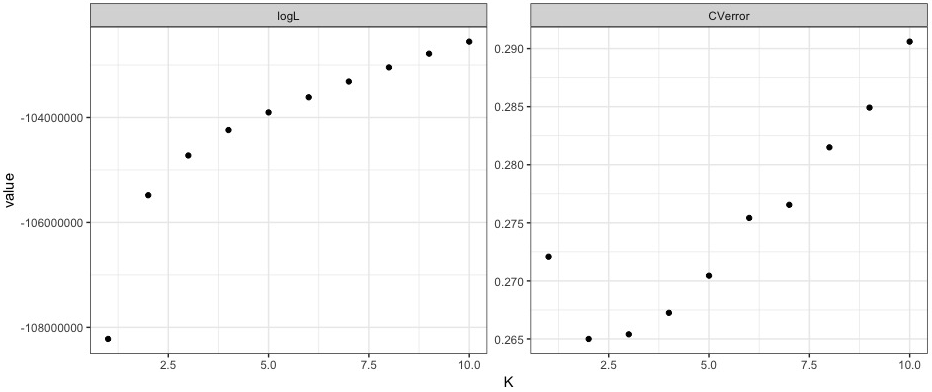


B)


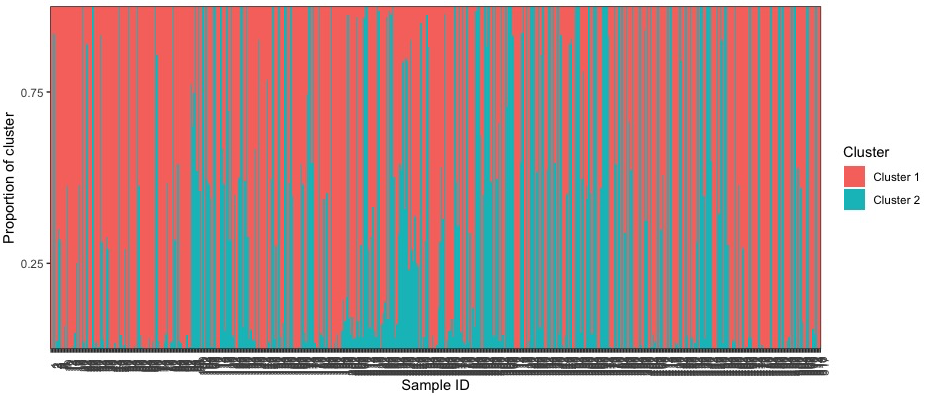


| 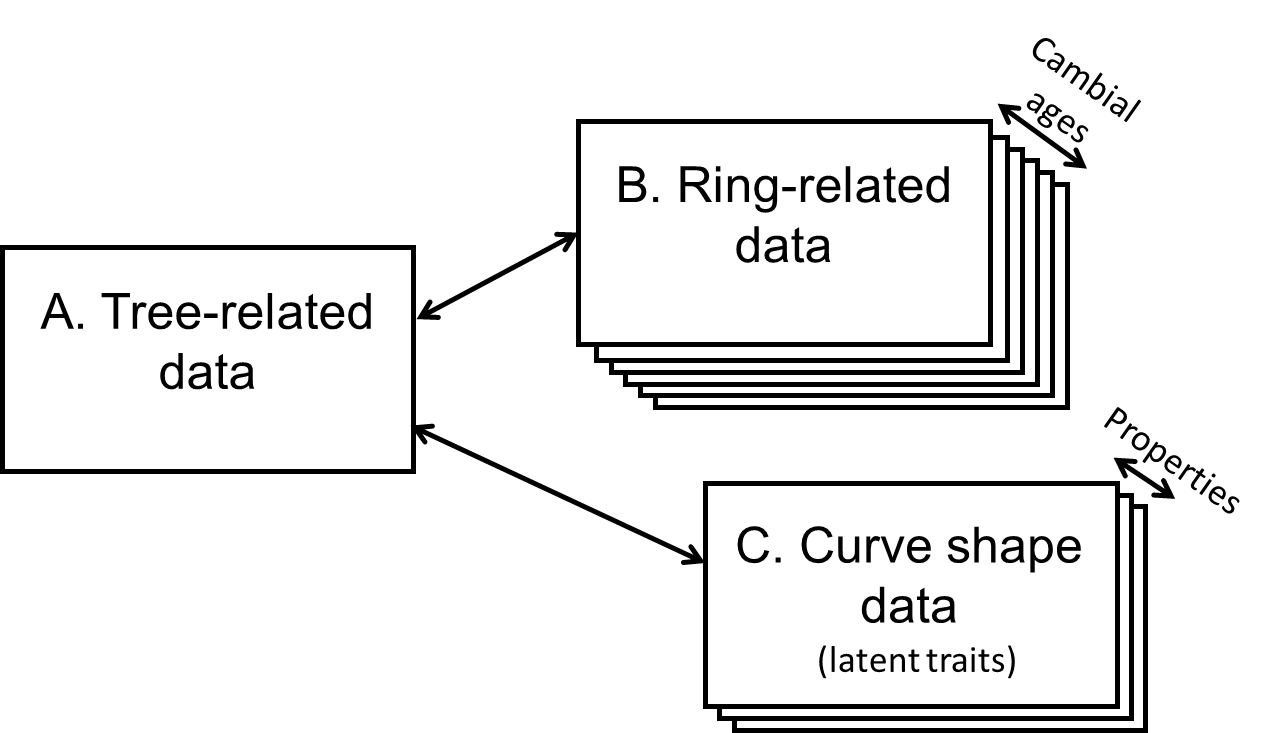 |
| --- |

Supplement: Supplementary file 1 — Figure S1. Phenotype trajectories representing the main traits. Figure S2. Significant contigs LD heatmap. Figure S3. ADMIXTURE plot of the entire population. Figure S4. Data are structured into three categories. [file TPJ-100-83-s001.docx]
