# Supplementary material for: Genome‐wide association study identified novel candidate loci affecting wood formation in Norway spruce
Source: Plant J. 2019 Jul 28;100(1):83–100. doi: 10.1111/tpj.14429 (PMC6852177; doi:10.1111/tpj.14429)
Supplement: Supplementary file 2 — Table S1. ConGenIE BLAST search of contigs with significant QTLs. Table S2. Ring‐related data (B): list of variables and examples of data. Table S3. Curve shape data (A): list of variables for each property and example of data. [file TPJ-100-83-s002.docx]

Association mapping identified candidate loci affecting wood formation in Norway spruce

Table S1 ConGenIE BLAST search of contigs with significant QTLs

| *Trait* | *Marker* | *Contig/Gene* | *Putative Genes* | | *GO* |
| --- | --- | --- | --- | --- | --- |
| RW | 11535 | MA_10694g0010 | -endoglucanase 11-like. Endohydrolysis of (1->4)-beta-D-glucosidic linkage in cellulose lichenin and cereal beta-D-glucans.  *Arabidopsis thaliana:*  Gene: [AT1G70710](http://atgenie.org/gene?id=AT1G70710)  Protein: glycosyl hydrolase 9B1  Populus:  Gene: [Potri.001G083200](http://popgenie.org/gene?id=Potri.001G083200)  Protein: ENDO-1,4-BETA-GLUCANASE | | [GO:0004553](http://amigo.geneontology.org/cgi-bin/amigo/term_details?term=GO:0004553) :- hydrolase activity  [GO:0005975](http://amigo.geneontology.org/cgi-bin/amigo/term_details?term=GO:0005975):- carbohydrate metabolism |
|  | 112391 | MA_879270g0010 | -DOX1 protein.  *Arabidopsis thaliana:*  Gene: AT3G01420  Protein: Alpha-dioxygenase 1, DIOX1  Populus:  Gene: [Potri.008G106400](http://popgenie.org/gene?id=Potri.008G106400)  Protein: putative alpha-dioxygenase. | | [GO:0004601](http://amigo.geneontology.org/cgi-bin/amigo/term_details?term=GO:0004601):- peroxidase activity  [GO:0006979](http://amigo.geneontology.org/cgi-bin/amigo/term_details?term=GO:0006979):- response to oxidative stress  [GO:0020037](http://amigo.geneontology.org/cgi-bin/amigo/term_details?term=GO:0020037):- heme binding  [GO:0055114](http://amigo.geneontology.org/cgi-bin/amigo/term_details?term=GO:0055114):- oxidation reduction |
|  | 165481 | MA_10434805g0020 | Proliferating cell nuclear antigen (PCNA) protein.  *Arabidopsis thaliana:*  Gene: [AT1G07370](http://atgenie.org/gene?id=AT1G07370)  Protein: Proliferating cell nuclear antigen  Populus:  Gene: [Potri.001G247700](http://popgenie.org/gene?id=Potri.001G247700)  Protein: similar to proliferating cell nuclear antigen | | [GO:0003677](http://amigo.geneontology.org/cgi-bin/amigo/term_details?term=GO:0003677):- DNA binding  [GO:0006275](http://amigo.geneontology.org/cgi-bin/amigo/term_details?term=GO:0006275):- regulation of DNA replication  [GO:0006281](http://amigo.geneontology.org/cgi-bin/amigo/term_details?term=GO:0006281):- DNA repair  [GO:0030337](http://amigo.geneontology.org/cgi-bin/amigo/term_details?term=GO:0030337):- DNA polymerase processivity factor activity  [GO:0043626](http://amigo.geneontology.org/cgi-bin/amigo/term_details?term=GO:0043626):- PCNA complex |
|  |  | | |  |  |
|  | 23808 | MA_20322g0010 | Plant-specific B3-DNA-binding domain protein (gene MTR_1g021440).  *Arabidopsis thaliana:*  Gene: VRN1 ([AT3G06220](http://atgenie.org/gene?id=AT3G06220))  Protein: AP2/B3-like transcriptional factor family protein  Populus:  Gene/locus: [Potri.014G036900](http://popgenie.org/gene?id=Potri.014G036900)  Protein: transcriptional factor B3 family protein; [ co-ortholog At4g33280] | | [GO:0003677](http://amigo.geneontology.org/cgi-bin/amigo/term_details?term=GO:0003677):- DNA binding  [GO:0006355](http://amigo.geneontology.org/cgi-bin/amigo/term_details?term=GO:0006355):- regulation of transcription, DNA-dependent |
|  | 112394 | MA_879384g0010 | E3 ubiquitin-protein ligase UBR3 (gene-Ubr3_2)  *Arabidopsis thaliana:*  Gene/Locus: At1g76250 ([AT1G76250.1](http://atgenie.org/transcript?id=AT1G76250.1))  Protein: At1g76250 (Uncharacterized)  Populus:  Gene/Locus: [Potri.005G249600](http://popgenie.org/gene?id=Potri.005G249600)  Protein: 27031567_peptide | | [GO:0008150](http://amigo.geneontology.org/cgi-bin/amigo/term_details?term=GO:0008150):- process  [GO:0012505](http://amigo.geneontology.org/cgi-bin/amigo/term_details?term=GO:0012505):- endomembrane system |
| TRW | 89295 | MA_214776g0010 | Serine/threonine-protein kinase (Os01g0689900)  Arabidopsis thaliana:  Gene/Locus: LRK10L-12 ([AT1G18390](http://atgenie.org/gene?id=AT1G18390))  Protein: Leaf Rust 10 Disease-Resistance Locus Receptor-Like Protein Kinase-like 1.2  Populus:  Gene/Locus: [Potri.004G096900](http://popgenie.org/gene?id=Potri.004G096900)  Protein: protein kinase activity | | [GO:0004672](http://amigo.geneontology.org/cgi-bin/amigo/term_details?term=GO:0004672):- protein kinase activity  [GO:0005524](http://amigo.geneontology.org/cgi-bin/amigo/term_details?term=GO:0005524):- ATP binding  [GO:0006468](http://amigo.geneontology.org/cgi-bin/amigo/term_details?term=GO:0006468):- protein amino acid phosphorylation |
|  | 33110 | MA_38472g0010 | Homeodomain protein HB2 from Picea abies.  Arabidopsis thaliana:  Gene/Locus: ANL2 ([AT4G00730](http://atgenie.org/gene?id=AT4G00730))  Protein Homeobox-leucine zipper protein ANTHOCYANINLESS  Populus:  Gene/Locus: Potri.014G075200  Protein: Homeodomain protein. | | [GO:0003700](http://amigo.geneontology.org/cgi-bin/amigo/term_details?term=GO:0003700):- transcription factor activity  [GO:0006355](http://amigo.geneontology.org/cgi-bin/amigo/term_details?term=GO:0006355):- regulation of transcription, DNA-dependent  [GO:0043565](http://amigo.geneontology.org/cgi-bin/amigo/term_details?term=GO:0043565):- sequence-specific DNA binding |
|  | 111057 | MA_817099g0010 | Predicted Senescence-associated protein  Arabidopsis thaliana:  Gene: AT3G51250  Protein: Senescence/dehydration-associated protein-related  Populus:  Gene: Potri.018G087500  Protein: Uncharacterised | | [GO:0003674](http://amigo.geneontology.org/cgi-bin/amigo/term_details?term=GO:0003674):- function  [GO:0005575](http://amigo.geneontology.org/cgi-bin/amigo/term_details?term=GO:0005575):- component  [GO:0008150](http://amigo.geneontology.org/cgi-bin/amigo/term_details?term=GO:0008150):- process |
| LRW | 164772 | MA_10434624g0020 | Pectinesterase from Picea sitchensis  Arabidopsis thaliana:  Gene/Locus: [AT1G53830](http://atgenie.org/gene?id=AT1G53830)  Protein: Pectin methylesterase 2  Populus:  Gene/Locus: [Potri.011G025400](http://popgenie.org/gene?id=Potri.011G025400)  Protein: Pectin methylesterase | | [GO:0004857](http://amigo.geneontology.org/cgi-bin/amigo/term_details?term=GO:0004857):- enzyme inhibitor activity  [GO:0005618](http://amigo.geneontology.org/cgi-bin/amigo/term_details?term=GO:0005618):- cell wall  [GO:0030599](http://amigo.geneontology.org/cgi-bin/amigo/term_details?term=GO:0030599):- pectinesterase activity  [GO:0042545](http://amigo.geneontology.org/cgi-bin/amigo/term_details?term=GO:0042545):- cell wall modification |
| NC | 104740 | MA_528074g0010 | LRR receptor-like kinase  Arabidopsis thaliana:  Gene/Locus: [AT5G20480](http://atgenie.org/gene?id=AT5G20480)  Protein: EF-TU receptor  Populus:  Gene/Locus: [Potri.013G020900](http://popgenie.org/gene?id=Potri.013G020900)  Protein: Similar to resistance protein. | | [GO:0004672](http://amigo.geneontology.org/cgi-bin/amigo/term_details?term=GO:0004672):- protein kinase activity  [GO:0005515](http://amigo.geneontology.org/cgi-bin/amigo/term_details?term=GO:0005515):- protein binding  [GO:0005524](http://amigo.geneontology.org/cgi-bin/amigo/term_details?term=GO:0005524):- ATP binding  [GO:0006468](http://amigo.geneontology.org/cgi-bin/amigo/term_details?term=GO:0006468):- protein amino acid phosphorylation |
| ENC | 98508 | MA_402880g0010 | UniProtKB-Putative serine incorporator.  Arabidopsis thaliana:  Gene/Locus: [AT4G13345](http://atgenie.org/gene?id=AT4G13345)  Protein: Serinc-domain containing serine and sphingolipid biosynthesis protein  Populus:  Gene/Locus: [Potri.018G076000](http://popgenie.org/gene?id=Potri.018G076000)  Protein: TMS membrane family protein | | [GO:0016020](http://amigo.geneontology.org/cgi-bin/amigo/term_details?term=GO:0016020):- membrane |
|  | 167610 | MA_10435406g0010 | Phosphoadenosine phosphosulfate reductase.  Arabidopsis thaliana:  Gene/Locus: AT3G59490  Protein: Uncharacterised  Populus  Gene/Locus: [Potri.017G028900](http://popgenie.org/gene?id=Potri.017G028900)  Protein: Uncharacterised | | [GO:0003674](http://amigo.geneontology.org/cgi-bin/amigo/term_details?term=GO:0003674):- function  [GO:0008150](http://amigo.geneontology.org/cgi-bin/amigo/term_details?term=GO:0008150):- process |
| TNC | 95870 | MA_346723g0010 | Protein detoxification Multi antimicrobial extrusion protein (domain MatE) identified in *Picea sitchensis*.  Arabidopsis thaliana: Non | |  |
|  | 126785 | MA_9447489g0010 | cDNA from *Oryza sativa* with a Pfam Peptidase domain. (Zinc carboxypeptidase enzyme).  Arabidopsis thaliana:  Gene/Locus: [AT5G42320](http://atgenie.org/gene?id=AT5G42320)  Protein: Zn-dependent exopeptidases superfamily protein  Populus:  Gene/Locus: [Potri.005G250300](http://popgenie.org/gene?id=Potri.005G250300)  Protein: Uncharacterised | | [GO:0004181](http://amigo.geneontology.org/cgi-bin/amigo/term_details?term=GO:0004181):- metallocarboxypeptidase activity  [GO:0006508](http://amigo.geneontology.org/cgi-bin/amigo/term_details?term=GO:0006508):- proteolysis and peptidolysis  [GO:0008270](http://amigo.geneontology.org/cgi-bin/amigo/term_details?term=GO:0008270):- zinc ion binding |
| LNC | 143628 | MA_10428744g0010 | DRY_EERY domain from the splicing factor SWAP (Suppressor of white apricot protein).  Arabidopsis thaliana:  Gene/Locus: [AT4G36980](http://atgenie.org/gene?id=AT4G36980)  Protein: Uncharacterised  Populus:  Gene/Locus: [Potri.007G044100](http://popgenie.org/gene?id=Potri.007G044100)  Protein: Uncharacterised | | [GO:0003674](http://amigo.geneontology.org/cgi-bin/amigo/term_details?term=GO:0003674):- function  [GO:0008150](http://amigo.geneontology.org/cgi-bin/amigo/term_details?term=GO:0008150):- process |
| EP | 16868 | MA_15729g0010 | DNA-3-methyladenine glycosylase II enzyme. A hydrolases enzyme.  Arabidopsis thaliana: Non | |  |

|  | 91242 | MA_246125g0010 | TIR/NBS/LRR domain present indicative of a disease resistance protein  Arabidopsis thaliana:  Gene/Locus: AT1G69550  Protein: Disease resistance protein (TIR-NBS-LRR class)  Populus:  Gene/Locus: Potri.005G031900  Protein: Uncharacterised | [GO:0005515](http://amigo.geneontology.org/cgi-bin/amigo/term_details?term=GO:0005515):- protein binding  [GO:0005524](http://amigo.geneontology.org/cgi-bin/amigo/term_details?term=GO:0005524):- ATP binding  [GO:0006915](http://amigo.geneontology.org/cgi-bin/amigo/term_details?term=GO:0006915):- apoptosis  [GO:0043531](http://amigo.geneontology.org/cgi-bin/amigo/term_details?term=GO:0043531):- ADP binding |
| --- | --- | --- | --- | --- |
| LP | 162397 | MA_10434007g0010 | Importin beta-like SAD2.  Arabidopsis thaliana:  Gene/Locus: [AT2G31660](http://atgenie.org/gene?id=AT2G31660)  Protein: ARM repeat superfamily protein  Populus:  Gene/Locus: [Potri.014G149600](http://popgenie.org/gene?id=Potri.014G149600)  Protein: Importin beta-2 subunit family protein | [GO:0006886](http://amigo.geneontology.org/cgi-bin/amigo/term_details?term=GO:0006886):- intracellular protein transport  [GO:0008565](http://amigo.geneontology.org/cgi-bin/amigo/term_details?term=GO:0008565):- protein transporter activity |
| TP | 101203 | MA_462319g0010 | Zein-binding domain identified within the sequence. | [GO:0003674](http://amigo.geneontology.org/cgi-bin/amigo/term_details?term=GO:0003674):- function  [GO:0005575](http://amigo.geneontology.org/cgi-bin/amigo/term_details?term=GO:0005575):- component  [GO:0008150](http://amigo.geneontology.org/cgi-bin/amigo/term_details?term=GO:0008150):- process |
| EP/LP | 51657 | MA_80954g0010 | PKS1  -  phytochrome kinase substrate 1.  Arabidopsis thaliana:  Gene/Locus: Swiss-Prot:Q9SWI1  Protein: phytochrome kinase substrate 1  Populus:  Gene/Locus: [Potri.005G090000](http://popgenie.org/gene?id=Potri.005G090000)  Protein: phytochrome kinase substrate-related |  |
|  | 60787 | MA_98424g0010 | OB-fold nucleic acid binding domain containing protein  Arabidopsis thaliana:  Gene/Locus: [AT1G07130](http://atgenie.org/gene?id=AT1G07130)  Protein: Nucleic acid-binding, OB-fold-like protein  Populus:  Gene/Locus: [Potri.005G212900](http://popgenie.org/gene?id=Potri.005G212900)  Protein: Uncharacterised | [GO:0003676](http://amigo.geneontology.org/cgi-bin/amigo/term_details?term=GO:0003676):- nucleic acid binding |
|  | 123639 | MA_8790100g0010 | ATPase family protein sequence  Arabidopsis thaliana:  Gene/Locus: [AT2G45500](http://atgenie.org/gene?id=AT2G45500)  Protein: AAA-type ATPase family protein  Populus:  Gene/Locus: Potri.014G071900  Protein: Uncharacterised | [GO:0005524](http://amigo.geneontology.org/cgi-bin/amigo/term_details?term=GO:0005524):- ATP binding  [GO:0006281](http://amigo.geneontology.org/cgi-bin/amigo/term_details?term=GO:0006281):- DNA repair  [GO:0006310](http://amigo.geneontology.org/cgi-bin/amigo/term_details?term=GO:0006310):- DNA recombination  [GO:0009378](http://amigo.geneontology.org/cgi-bin/amigo/term_details?term=GO:0009378):- Holliday junction helicase activity |

|  | 59480 | MA_96191g0010 | | Glycosyltransferase from *Picea sitchensis.*  Arabidopsis thaliana:  Gene/Locus: [AT4G34135](http://atgenie.org/gene?id=AT4G34135)  Protein: UDP-glucosyltransferase 73B2  Populus:  Gene/Locus: Potri.001G303700  Protein: Uncharacterised | [GO:0008152](http://amigo.geneontology.org/cgi-bin/amigo/term_details?term=GO:0008152):- metabolism  [GO:0016758](http://amigo.geneontology.org/cgi-bin/amigo/term_details?term=GO:0016758):- transferase activity, transferring hexosyl groups |
| --- | --- | --- | --- | --- | --- |
|  | 117333 | MA_1045136g0010 | | TIR-NBS-LRR domain present associated with disease resistance.Arabidopsis thaliana:Gene/Locus: AT5G46270Protein: Disease resistance protein (TIR-NBS-LRR class) familyPopulus:Gene/Locus: Potri.013G097800Protein: Uncharacterised | [GO:0004888](http://amigo.geneontology.org/cgi-bin/amigo/term_details?term=GO:0004888):- transmembrane receptor activity  [GO:0005515](http://amigo.geneontology.org/cgi-bin/amigo/term_details?term=GO:0005515):- protein binding  [GO:0005524](http://amigo.geneontology.org/cgi-bin/amigo/term_details?term=GO:0005524):- ATP binding  [GO:0005622](http://amigo.geneontology.org/cgi-bin/amigo/term_details?term=GO:0005622):- intracellular  [GO:0006915](http://amigo.geneontology.org/cgi-bin/amigo/term_details?term=GO:0006915):- apoptosis  [GO:0007165](http://amigo.geneontology.org/cgi-bin/amigo/term_details?term=GO:0007165):- signal transduction  [GO:0031224](http://amigo.geneontology.org/cgi-bin/amigo/term_details?term=GO:0031224):- intrinsic to membrane  [GO:0043531](http://amigo.geneontology.org/cgi-bin/amigo/term_details?term=GO:0043531):- ADP binding  [GO:0045087](http://amigo.geneontology.org/cgi-bin/amigo/term_details?term=GO:0045087):- innate immune response |
|  | 101118 | MA_460877g0010 | | Toll/interleukin-1 receptor (TIR). An intracellular signaling domain Arabidopsis thaliana: Gene/Locus: [AT4G12010](http://atgenie.org/gene?id=AT4G12010) Protein: Disease resistance protein (TIR-NBS-LRR class)Populus: Gene/Locus: [Potri.017G105500.1](http://popgenie.org/transcript?id=Potri.017G105500.1) Protein: Uncharacterised | [GO:0004888](http://amigo.geneontology.org/cgi-bin/amigo/term_details?term=GO:0004888):- transmembrane receptor activity  [GO:0005515](http://amigo.geneontology.org/cgi-bin/amigo/term_details?term=GO:0005515):- protein binding  [GO:0005524](http://amigo.geneontology.org/cgi-bin/amigo/term_details?term=GO:0005524):- ATP binding  [GO:0005622](http://amigo.geneontology.org/cgi-bin/amigo/term_details?term=GO:0005622):- intracellular  [GO:0006915](http://amigo.geneontology.org/cgi-bin/amigo/term_details?term=GO:0006915):- apoptosis  [GO:0007165](http://amigo.geneontology.org/cgi-bin/amigo/term_details?term=GO:0007165):- signal transduction  [GO:0031224](http://amigo.geneontology.org/cgi-bin/amigo/term_details?term=GO:0031224):- intrinsic to membrane  [GO:0043531](http://amigo.geneontology.org/cgi-bin/amigo/term_details?term=GO:0043531):- ADP binding  [GO:0045087](http://amigo.geneontology.org/cgi-bin/amigo/term_details?term=GO:0045087):- innate immune response |
| WD | 167610 | MA_10435406g0010 | | Phosphoadenosine phosphosulfate reductase. Gene cysH_2Arabidopsis thaliana:Gene/Locus: [AT3G59490](http://atgenie.org/gene?id=AT3G59490)Protein: UncharacterisedPopulus:Gene/Locus: [Potri.017G028900](http://popgenie.org/gene?id=Potri.017G028900)Protein: Uncharacterised | [GO:0003674](http://amigo.geneontology.org/cgi-bin/amigo/term_details?term=GO:0003674):- function  [GO:0008150](http://amigo.geneontology.org/cgi-bin/amigo/term_details?term=GO:0008150):- process |
|  | 30469 | MA_33109g0010 | | -60S ribosomal protein L10 from Pinus taedaArabidopsis thaliana:Gene/Locus: [AT1G66580](http://atgenie.org/gene?id=AT1G66580)Protein: Senescence associated gene 24Populus:Gene/Locus: [Potri.013G159600](http://popgenie.org/gene?id=Potri.013G159600)Protein: Similar to 60S ribosomal protein L10 (Wilm's tumor suppressor protein homolog) | [GO:0003735](http://amigo.geneontology.org/cgi-bin/amigo/term_details?term=GO:0003735):- structural constituent of ribosome  [GO:0005840](http://amigo.geneontology.org/cgi-bin/amigo/term_details?term=GO:0005840):- ribosome  [GO:0006412](http://amigo.geneontology.org/cgi-bin/amigo/term_details?term=GO:0006412):- protein biosynthesis |
|  | 157442 | MA_10432646g0010 | | Putative uncharacterized protein from Picea sitchensis.Arabidopsis thaliana:Gene/Locus: [AT1G17130](http://atgenie.org/gene?id=AT1G17130)Protein: Family of unknown function (DUF572)Populus:Gene/Locus: [Potri.001G377800](http://popgenie.org/gene?id=Potri.001G377800)Protein: Uncharacterised | [GO:0003674](http://amigo.geneontology.org/cgi-bin/amigo/term_details?term=GO:0003674):- function  [GO:0005575](http://amigo.geneontology.org/cgi-bin/amigo/term_details?term=GO:0005575):- component  [GO:0008150](http://amigo.geneontology.org/cgi-bin/amigo/term_details?term=GO:0008150):- process |
| EWD | 167610 | MA_10435406g0010 | | -Phosphoadenosine phosphosulfate reductase. Gene cysH_2Arabidopsis thaliana:Gene/Locus: [AT3G59490](http://atgenie.org/gene?id=AT3G59490)Protein: UncharacterisedPopulus:Gene/Locus: [Potri.017G028900](http://popgenie.org/gene?id=Potri.017G028900)Protein: Uncharacterised | [GO:0003674](http://amigo.geneontology.org/cgi-bin/amigo/term_details?term=GO:0003674):- function  [GO:0008150](http://amigo.geneontology.org/cgi-bin/amigo/term_details?term=GO:0008150):- process |
|  | 23798 | MA_20321g0010 | | -Serine/threonine-protein phosphataseArabidopsis thaliana:Gene/Locus: [AT1G08420](http://atgenie.org/gene?id=AT1G08420)Protein: BRI1 suppressor 1 (BSU1)-like 2Populus:Gene/Locus: [Potri.009G016900](http://popgenie.org/gene?id=Potri.009G016900)Protein: Similar to kelch repeat-containing protein; serine/threonine phosphoesterase family protein | [GO:0005515](http://amigo.geneontology.org/cgi-bin/amigo/term_details?term=GO:0005515):- protein binding  [GO:0016787](http://amigo.geneontology.org/cgi-bin/amigo/term_details?term=GO:0016787):- hydrolase activity |
|  | 70955 | MA_118446g0010 | | -DICER-LIKE3 (DCL3b) protein from Pinus tabuliformis. |  |
| TWD | 131698 | MA_10235390g0010 | | -DNA mismatch repair protein MSH5 (gene- MSH5).Arabidopsis thaliana:Gene/Locus: [AT3G20475](http://atgenie.org/gene?id=AT3G20475)Protein: MUTS-homologue 5 (DNA mismatch repair protein MSH5)Populus:Gene/Locus: [Potri.011G089500](http://popgenie.org/gene?id=Potri.011G089500)Protein: Uncharacterised | [GO:0005524](http://amigo.geneontology.org/cgi-bin/amigo/term_details?term=GO:0005524):- ATP binding  [GO:0006298](http://amigo.geneontology.org/cgi-bin/amigo/term_details?term=GO:0006298):- mismatch repair  [GO:0030983](http://amigo.geneontology.org/cgi-bin/amigo/term_details?term=GO:0030983):- mismatched DNA binding |
|  | 160208 | MA_10433411g0010 | | -Heterotrimeric guanine nucleotide-binding protein subunit beta (gene G-beta)Arabidopsis thaliana:Gene/Locus: [AT1G18080](http://atgenie.org/gene?id=AT1G18080)Protein: Transducin/WD40 repeat-like superfamily proteinPopulus:Gene/Locus: [Potri.012G049600](http://popgenie.org/gene?id=Potri.012G049600)Protein: Uncharacterised | [GO:0005515](http://amigo.geneontology.org/cgi-bin/amigo/term_details?term=GO:0005515):- protein binding |
|  | 89044 | MA_212523g0010 | | -Kinesin-related protein 13 (gene-L484_021891). | [PF01846](http://pfam.xfam.org/family/PF01846):- FF domain |
| LWD | 43797 | MA_62987g0010 | | -Protein binding protein, with zinc ion binding properties. Gene RCOM_0475450. |  |
|  | 165481 (Width) | MA_10434805g0010 | | Proliferating cell nuclear antigen protein domain.*Arabidopsis thaliana:*Gene: [AT1G07370](http://atgenie.org/gene?id=AT1G07370)Protein: Proliferating cell nuclear antigenPopulus:Gene: [Potri.001G247700](http://popgenie.org/gene?id=Potri.001G247700)Protein: similar to proliferating cell nuclear antigen | [GO:0003677](http://amigo.geneontology.org/cgi-bin/amigo/term_details?term=GO:0003677):- DNA binding  [GO:0006275](http://amigo.geneontology.org/cgi-bin/amigo/term_details?term=GO:0006275):- regulation of DNA replication  [GO:0006281](http://amigo.geneontology.org/cgi-bin/amigo/term_details?term=GO:0006281):- DNA repair  [GO:0030337](http://amigo.geneontology.org/cgi-bin/amigo/term_details?term=GO:0030337):- DNA polymerase processivity factor activity  [GO:0043626](http://amigo.geneontology.org/cgi-bin/amigo/term_details?term=GO:0043626):- PCNA complex |
|  | 171223 | MA_10436058g0010 | | Gamma-tubulin complex component. Gamma-tubulin complex is necessary for microtubule nucleation at the centrosome.*Arabidopsis thaliana:*Gene:AT1G20570Protein: Spc97 / Spc98 family of spindle pole body (SBP) componentPopulus:Gene: [Potri.016G000100](http://popgenie.org/gene?id=Potri.016G000100)Protein: Uncharacterised | [GO:0000226](http://amigo.geneontology.org/cgi-bin/amigo/term_details?term=GO:0000226):- microtubule cytoskeleton organization and biogenesis  [GO:0000922](http://amigo.geneontology.org/cgi-bin/amigo/term_details?term=GO:0000922):- spindle pole  [GO:0005815](http://amigo.geneontology.org/cgi-bin/amigo/term_details?term=GO:0005815):- microtubule organizing center |
| Growth/Density (MI) | 166235 | | MA_10435002g0010 | (UniProt BLAST)Maize: ZEAMMB73_Zm00001d018274Description: Isoleucine-tRNA ligase chloroplastic/mitochondrial |  |
|  | 61096 | | MA_99004g0010 | Populus (Trans): [Potri.017G025900](http://popgenie.org/gene?id=Potri.017G025900)Arabidopsis: AT4G37650Description: GRAS family transcription factor | [GO:0003700](http://amigo.geneontology.org/cgi-bin/amigo/term_details?term=GO:0003700):- transcription factor activity  [GO:0005515](http://amigo.geneontology.org/cgi-bin/amigo/term_details?term=GO:0005515):- protein binding  [GO:0005634](http://amigo.geneontology.org/cgi-bin/amigo/term_details?term=GO:0005634):- nucleus  [GO:0007049](http://amigo.geneontology.org/cgi-bin/amigo/term_details?term=GO:0007049):- cell cycle  [GO:0008356](http://amigo.geneontology.org/cgi-bin/amigo/term_details?term=GO:0008356):- asymmetric cytokinesis  [GO:0009956](http://amigo.geneontology.org/cgi-bin/amigo/term_details?term=GO:0009956):- radial pattern formation  [GO:0032350](http://amigo.geneontology.org/cgi-bin/amigo/term_details?term=GO:0032350) |
|  | 67181 | | MA_109804g0010 | Populus (Trans): [Potri.011G122100.1](http://popgenie.org/transcript?id=Potri.011G122100.1)Arabidopsis: AT4G27450Description: Aluminium induced protein with YGL and LRDR motifs | [GO:0003674](http://amigo.geneontology.org/cgi-bin/amigo/term_details?term=GO:0003674):- function  [GO:0005634](http://amigo.geneontology.org/cgi-bin/amigo/term_details?term=GO:0005634):- nucleus  [GO:0005829](http://amigo.geneontology.org/cgi-bin/amigo/term_details?term=GO:0005829):- cytosol  [GO:0005886](http://amigo.geneontology.org/cgi-bin/amigo/term_details?term=GO:0005886):- plasma membrane  [GO:0008150](http://amigo.geneontology.org/cgi-bin/amigo/term_details?term=GO:0008150):- process |
|  | 1401 | | MA_1378g0010 | (UniProt BLAST)Populus: POPTR_0016s06970gArabidposis: AXX17_At3g05280Description: Protein virilizer may be involved in mRNA splicing regulation. |  |
|  | 138744 | | MA_10427214g0010 | Uncharacterized |  |
|  | 162397 | | MA_10434007g0010 | Populus: [Potri.014G149600.1](http://popgenie.org/transcript?id=Potri.014G149600.1)Arabidopsis: AT2G31660Description: ARM repeat superfamily protein. | [GO:0006886](http://amigo.geneontology.org/cgi-bin/amigo/term_details?term=GO:0006886):- intracellular protein transport  [GO:0008565](http://amigo.geneontology.org/cgi-bin/amigo/term_details?term=GO:0008565):- protein transporter activity |
|  | 21921 | | MA_19222g0010 | (UniProt BLAST)Uncharacterized-ADP (NB-ARC domain) (Pfam identification) binding protein domain in Picea sitchensis |  |

Table S2: Ring-related data (B): List of variables and examples of data

Example of data from the first trees and rings listed

Table S3: Curve shape data (A): List of variables for each property and example of data

| Field name | Description |
| --- | --- |
| TreeN | Tree number |
| Mum_id_N | Mothers genotype id |
| Property | Wood property |
| Int | Intercept |
| Slo | Slope |
| k1 | Location of first knee of spline function |
| k2 | Location of second knee of spline function |

Example of data from the first families listed
