# Supplementary material for: Genome‐wide association study identified novel candidate loci affecting wood formation in Norway spruce
Source: Plant J. 2019 Jul 28;100(1):83–100. doi: 10.1111/tpj.14429 (PMC6852177; doi:10.1111/tpj.14429)
Supplement: Supplementary file 3 — Methods S1. PVE evaluation of a QTL. Methods S2. Trait data set used for GWAS identification of novel candidate loci affecting number of tracheids formed, radial growth, density, stiffness and mass at breast height of young Norway spruce. [file TPJ-100-83-s003.docx]

Association mapping identified candidate loci affecting wood formation in Norway spruce

**Methods S1** PVE evaluation of a QTL

PVE evaluation:

where the penalty function has a separate weight $\omega_{j}=\frac{1}{|\hat{\alpha}_{\mathrm{LASSO}j}|+1/n}$ ($\hat{\alpha}_{\mathrm{LASSO}j}$ represents the initial LASSO estimates from (3)) for each independent SNP, and the rest of the parameters are defined in the same way as in the standard LASSO regression (3). By the aid of the SNP specific tuning factor, adaptive LASSO should provide un-biased estimates to the effect size and PVE of a QTL.

**Methods S2** Trait data set used for GWAS identification of novel candidate loci affecting number of tracheids formed, radial growth, density, stiffness and mass at breast height of young Norway spruce

Measurements and refinement of data

The cores were prepared for and analysed at Innventia, now part of RISE Bioeconomy, with its SilviScan instrument (Evans 1994, 2005). Detailed information was compiled on radial variations from pith to bark in growth, tracheid and wood traits, followed by several steps of evaluations: All annual rings and their compartments of earlywood (EW), transitionwood (TW) and latewood (LW) were identified. Further traits such as number of tracheids formed were derived and averages were calculated for rings and their parts. The data were organised in a database, providing structured information for the next steps of evaluations addressing different topics.

The transitionwood of each annual ring, the zone with varying width and character under strong influence from weather between the pronounced earlywood and latewood parts of more stable properties, was isolated using the density-based “20-80” definition. This provided more stable information on properties of the pronounced EW and LW, which are expected to relate stronger to the genes. More details and motives for this are given in Lundqvist et al 2018.

Traits investigated

The traits emphasised in Baison et al 2018 relate to growth, wood density and wood mass formed at breast height, complemented with wood stiffness (MOE), all aspects of major importance for industrial use in structural timber products.

Structure of data

The data are structured into three categories according to Fig S4.

|  |
| --- |

1. Tree-related data: For each tree an identity number, its experimental site and family, as well as the mass-index at breast height calculated for the tree
2. Ring-related data: For each ring the tree identity number and the number of the ring (cambial age), its radial location and averages for rings and their parts of the traits in Table 1.
3. Curve shape data (latent variables): Intercept, slope and locations of knots of spline functions

In Tables 2-3, all variables included in the three parts are specified and exemplified with data for the first trees, rings and families, respectively, listed for each type of data.

| Table 3: Tree-related data (A): List of variables and example of data  Examples of data, the first trees in the table   \| Field name \| Description \| \| --- \| --- \| \| TreeN \| Tree number \| \| Trial_id_short \| Trial identity \| \| Mum_id_N \| Mothers genotype id \| \| Mass_index \| Relative cross-sectional mass at breast height: (Cross-sectional_denisty x Cross-sectional_area)/(Mean Cross-sectional_ density of all samples x Mean Cross-sectional area of all samples) \|  \| TreeN* \| Trial_id_short \| Mum_id_N \| Mass_index \| \| --- \| --- \| --- \| --- \| \| 1 \| 2 \| 318 \| 1,49 \| \| 4 \| 2 \| 123 \| 1,21 \| \| 5 \| 2 \| 443 \| 0,97 \| \| 6 \| 2 \| 423 \| 0,44 \| \| 7 \| 2 \| 501 \| 1,53 \| \| 9 \| 2 \| 256 \| 1,29 \| \| 10 \| 2 \| 315 \| 0,92 \| \| 12 \| 2 \| 435 \| 0,90 \| \| 13 \| 2 \| 66 \| 1,24 \| \| 14 \| 2 \| 413 \| 1,03 \| |
| --- | --- | --- | --- | --- | --- | --- | --- | --- | --- | --- | --- | --- | --- | --- | --- | --- | --- | --- | --- | --- | --- | --- | --- | --- | --- | --- | --- | --- | --- | --- | --- | --- | --- | --- | --- | --- | --- | --- | --- | --- | --- | --- | --- | --- | --- | --- | --- | --- | --- | --- | --- | --- | --- | --- |
| etc. |

* TreeN is a running number for the trees initially selected as candidates for sampling, with some extra trees per family to choose the 6 trees per trail aimed for, as some trees might be unsuitable (dead, broken, etc.). Thus, the absence of the trees numbered 2, 3, 8 and 11 in the table has no relevance for the study.
