# Supplementary material for: Genome‐wide association study identified novel candidate loci affecting wood formation in Norway spruce
Source: Plant J. 2019 Jul 28;100(1):83–100. doi: 10.1111/tpj.14429 (PMC6852177; doi:10.1111/tpj.14429)
Supplement: Supplementary file 4 [file TPJ-100-83-s004.docx]

Association mapping identified candidate loci affecting wood formation in Norway spruce

**Figure S1.** Phenotype Trajectories representing the main traits (Density, Annual Ring Width, Total Number of Cells and Early/Latewood Percentage.

**Figure S2.** Significant Contigs LD Heatmap.

**Figure S3.** ADMIXTURE plot of the entire population.

**Figure S4.** The data are structured into three categories.

**Table S1.** ConGenIE BLAST search of contigs with significant QTLs.

**Table S2.** Ring-related data (B): List of variables and examples of data.

**Table S3.** Curve shape data (A): List of variables for each property and example of data.

**Methods S1.** PVE evaluation of a QTL.

**Methods S2.** Trait data set used for GWAS identification of novel candidate loci affecting number of tracheids formed, radial growth, density, stiffness and mass at breast height of young Norway spruce.
